# Cranial Ontogeny in *Stegoceras validum* (Dinosauria: Pachycephalosauria): A Quantitative Model of Pachycephalosaur Dome Growth and Variation

**DOI:** 10.1371/journal.pone.0021092

**Published:** 2011-06-29

**Authors:** Ryan K. Schott, David C. Evans, Mark B. Goodwin, John R. Horner, Caleb Marshall Brown, Nicholas R. Longrich

**Affiliations:** 1 Department of Ecology and Evolutionary Biology, University of Toronto, Toronto, Ontario, Canada; 2 Royal Ontario Museum, Toronto, Ontario, Canada; 3 Museum of Paleontology, University of California, Berkeley, California, United States of America; 4 Museum of the Rockies, Montana State University, Bozeman, Montana, United States of America; 5 Department of Geology, Yale University, New Haven, Connecticut, United States of America; Raymond M. Alf Museum of Paleontology, United States of America

## Abstract

Historically, studies of pachycephalosaurs have recognized plesiomorphically flat-headed taxa and apomorphically domed taxa. More recently, it has been suggested that the expression of the frontoparietal dome is ontogenetic and derived from a flat-headed juvenile morphology. However, strong evidence to support this hypothesis has been lacking. Here we test this hypothesis in a large, stratigraphically constrained sample of specimens assigned to *Stegoceras validum*, the best known pachycephalosaur, using multiple independent lines of evidence including conserved morphology of ornamentation, landmark-based allometric analyses of frontoparietal shape, and cranial bone histology. New specimens show that the diagnostic ornamentation of the parietosquamosal bar is conserved throughout the size range of the sample, which links flat-headed specimens to domed *S. validum*. High-resolution CT scans of three frontoparietals reveal that vascularity decreases with size and document a pattern that is consistent with previously proposed histological changes during growth. Furthermore, aspects of dome shape and size are strongly correlated and indicative of ontogenetic growth. These results are complementary and strongly support the hypothesis that the sample represents a growth series of a single taxon. Cranial dome growth is positively allometric, proceeds from a flat-headed to a domed state, and confirms the synonymy of *Ornatotholus browni* as a juvenile *Stegoceras*. This dataset serves as the first detailed model of growth and variation in a pachycephalosaur. Flat-headed juveniles possess three characters (externally open cranial sutures, tuberculate dorsal surface texture, and open supratemporal fenestrae) that are reduced or eliminated during ontogeny. These characters also occur in putative flat-headed taxa, suggesting that they may also represent juveniles of domed taxa. However, open cranial sutures and supratemporal fenestrae are plesiomorphic within Ornithischia, and thus should be expected in the adult stage of a primitive pachycephalosaur. Additional lines of evidence will be needed to resolve the taxonomic validity of flat-headed pachycephalosaur taxa.

## Introduction

Pachycephalosauria is a clade of small- to medium-sized herbivorous dinosaurs that inhabited North America and Asia during the Late Cretaceous [1]. Most pachycephalosaur species are known primarily from cranial material, and most specimens consist only of the thickened frontoparietal that is characteristic of the group. Traditionally, two types of pachycephalosaurs have been recognized: those with thickened, but relatively flat frontoparietals and those with frontoparietals that are thickened to form a dome. These two morphological types have been recognized as separate clades: the flat-headed Homalocephalidae and the domed Pachycephalosauridae [2]–[4]. Other studies, including most recent phylogenetic analyses, do not recognize Homalocephalidae and instead the flat-headed taxa are found to form successive sister taxa to Pachycephalosauridae, which remain a monophyletic group [1], [5]–[10]. Despite this, placement of flat-headed and domed pachycephalosaurs into separate taxa may not always be accurate.

The first pachycephalosaur frontoparietal found resembling a flat-headed condition, AMNH 5450, consists of an incipiently domed frontoparietal. This specimen, characterized by the separation of low frontal and parietal domes by a shallow transverse depression and the large size of the open supratemporal fenestrae, was originally referred to *Stegoceras validum* by Galton [11]. Following Brown and Schlaikjer [12], Galton [11] suggested that there was sexual variation in the degree of doming and hypothesized that AMNH 5450, with its relatively low dome, represents a female morph of *S. validum*. Subsequently, Wall and Galton [13] transferred AMNH 5450 to a new species, *S. browni*, based on a lack of overlap with *S. validum* in three frontoparietal indices (width vs. length, height vs. length, and height vs. width) described by Brown and Schlaikjer [12]. Later, Galton and Sues [14] erected a new genus, *Ornatotholus*, for *S. browni*. This was based largely on the lack of a dome in AMNH 5450, which was similar in length to a domed specimen of *S. validum* (CMN 138), and on the relatively larger diameter of the open supratemporal fenestrae of AMNH 5450 compared to *S. validum*. Galton and Sues [14] described and referred additional isolated flat frontals and parietals from the Campanian of Alberta to *Ornatotholus* in further support of the taxonomic distinction of *O. browni* from *Stegoceras*. Galton and Sues [14] did not think that such pronounced differences in doming could be due to individual or sexual variation alone, and considered the many small tubercles that cover the dorsal surface of *O. browni* to be a unique diagnostic character.

Goodwin et al. [15] suggested that inflation of the frontoparietal dome was an ontogenetic feature, based on the cranial histology of a specimen referred to *Stegoceras validum* (MOR 295) that possessed highly vascularized, fast-growing primary bone consistent with an increase in doming with ontogenetic age. The putatively diagnostic characters of *Ornatotholus browni* identified by previous authors [11], [13], [14], such as the shallow transverse depression and large supratemporal fenestrae, were hypothesized to be ontogenetically variable within *Stegoceras* [15]. Based on this, Goodwin et al. [15] suggested that *O. browni* represents a juvenile ontogenetic stage of *Stegoceras.*

In accord with Goodwin et al. [15], Williamson and Carr [10] constructed a hypothetical growth series for *Stegoceras validum* in which they posited that *Ornatotholus browni* might represent a juvenile of *S. validum*. However, they admitted that they could not demonstrate synonymy because *O. browni* lacks any characters that would allow it to be referred to *S. validum*. Diagnostic characters of the frontoparietal dome appear only to develop at later ontogenetic stages in *S. validum* [10]. Consequently, Williamson and Carr [10] considered *O. browni* to be Pachycephalosauridae incertae sedis. Sullivan [9] formally synonymized *O. browni* and *S. validum* and also referred numerous isolated flat frontals and parietals to the latter taxon. Sullivan [9] did not address the issues raised by Williamson and Car [10], and his reasons for synonymy are unclear. In their reviews of Pachycephalosauria, Sereno [8] and Maryańska et al. [1] recognized *O. browni* as a distinct taxon, but provided no justification or comments on its ontogenetic status. A summary of the taxonomic history, diagnostic characters, and morphological interpretations of AMNH 5450 is provided in Table 1.

**Table 1:** Reference and taxonomic assignment of AMNH 5450, the holotype of *Ornatotholus browni* with accompanying diagnostic characters and additional comments from this study.

Reference	Taxonomic Assignment	Diagnosis and/or Description	Comments (this study)
Galton (1971)	*Stegoceras validum*	Female dimorph and most primitive pachycephalosaurid	Gender assignment untestable and primitive condition is unsupported morphologically
Wall and Galton (1979)	*Stegoceras browni*	Skull roof thick, low fp dome, and a shallow depression separates frontal from slightly lower dome on parietal	Diagnosis based on ontogenetic features not present in subadult–adult *S. validum*
Galton and Sues (1983)	*Ornatotholus browni*	Low fp dome, frontal and parietal separated by a shallow transverse depression, parietal slightly lower than frontal, and stf much larger than in *S. validum*	Diagnosis for genus is defined by transitional ontogenetic characters absent in adult *Stegoceras* sp.
Sues and Galton (1987)	*Ornatotholus browni*	Low fp dome, in holotype frontal and parietal dome divided by a shallow transverse depression; fp covered by prominent tubercles dorsally	Revised diagnosis defined by transitional ontogenetic characters absent in adult *Stegoceras* sp.
Goodwin et al. (1998)	*Stegoceras validum* (juvenile)	Reinterpret *O. browni* as a juvenile *S. validum* based on its flat, uninflated fp, open midline frontal suture and fp suture dorsally, open stf, holotype is 30% smaller than domed *S. validum* specimens	First reappraisal of *O. browni* as a juvenile *S. validum*
Williamson and Carr (2002)	*Ornatotholus browni* (nomen dubium)	*O. browni* represents a possible juvenile *S. validum*	Confirmed in this study
Sullivan (2003)	*Ornatotholus browni* ( = *S. validum*)	*O. browni* is a synonym of *S. validum*	Confirmed in this study
Maryańska et al. (2004)	*Ornatotholus browni*	*O. browni* recognized as a distinct taxon	Does not discuss ontogenetic or taxonomic status of *O. browni*
This study	*Stegoceras validum* (juvenile)	Allometry and histology confirm juvenile status of the holotype of *O. browni* and synonymy with *S. validum* (Sullivan, 2003)	AMNH 5450 is an early ontogenetic stage of *S. validum*

Abbreviations: **fp**, frontoparietal; **stf**, supratemporal fenestrae.

As recognized by Williamson and Carr [10], pachycephalosaurs with flat-headed frontoparietals attributed to *Ornatotholus*, and subsequently considered juvenile specimens of *Stegoceras validum*, have presented a taxonomic problem because they exhibit the plesiomorphic condition present in putatively distinct flat-headed pachycephalosaurs, such as *Wannanosaurus*, *Goyocephale*, and *Homalocephale*, and lack diagnostic characters that would link them definitively to adult stages of any given taxon. Furthermore, authors have begun to question the taxonomic validity of these putatively primitive flat-headed taxa and suggested that they may represent juveniles of previously known taxa [16], [17]. Other authors maintain the view that at least some of these taxa are distinct, but are represented only by juveniles [18], [19], or possibly represent paedomorphic adult morphologies [20]. A more complete understanding of pachycephalosaur ontogeny and variation is required to resolve these outstanding questions that impact our understanding of pachycephalosaur biodiversity and relative abundance.

Here we test the hypothesis that *Stegoceras validum* developed ontogenetically from a flat-headed morphology to a domed morphology using multiple independent lines of evidence on a large, stratigraphically constrained sample of specimens referred to *S. validum* [9] from the Belly River Group of Alberta (predominantly Dinosaur Park Formation). The lines of evidence used include traditional comparative morphology, with description of the first definitively flat-headed specimens of *S. validum* that preserve diagnostic squamosal ornamentation, quantitative assessment of frontoparietal allometry, and both qualitative and quantitative analysis of bone histology through the use of high-resolution X-ray computed tomography (HRCT). This study provides strong evidence linking flat-headed and domed morphologies within an ontogenetic series of a single taxon, and serves as the most complete model of growth and variation in a pachycephalosaur.

### Frontoparietal Allometry

Chapman et al. [21] were the first to attempt to quantify dome allometry in pachycephalosaurs. They performed a series of bivariate comparisons using reduced major axis (RMA) regression. With respect to the growth of the frontoparietal dome, these authors compared ‘dome length’ and ‘dome thickness’ and concluded that the dome thickened isometrically with increased length [21]. Their study was based on a sample of frontoparietal domes and incomplete skulls from Alberta, Canada, that they identified as *Stegoceras validum* (except for *S. edmontonense* from the Horseshoe Canyon Formation, the poorly preserved holotype frontoparietal of *Gravitholus albertae*, and AMNH 5450, the holotype of *Ornatotholus*, which they recognized as a distinct species) [21]. On the basis of their morphometric analysis, they interpreted their sample of ‘*S. validum*’ as a single, sexually dimorphic species (excluding *S. edmontonense*, *Gravitholus*, and *Ornatotholus*) [21]. The ‘*S. validum*’ dataset of Chapman et al. [21] is now known to include specimens from three different formations (Foremost, Oldman, and Dinosaur Park) that are derived from sediments that encompass at least five million years of time [22], and subsequent taxonomic studies now recognize up to four different taxa within this sample: *Stegoceras validum*, *Prenocephale brevis*, *Hanssuesia sternbergi,* and *Colepiocephale lambei* [9]. Thus, the results of the Chapman et al. [21] study likely reflect a combination of interspecific differences and intraspecific allometry that may not adequately describe the growth dynamics in an individual species. In addition, there were several problems with their analytical approach, notably the measurement indices used by Chapman et al. [21] were not based on homologous landmarks and thus measurements between specimens are not comparable [23].

The large sample of specimens referred to *Stegoceras validum* [9], including flat-headed specimens with diagnostic squamosal ornamentation, represents the most complete sample of ontogenetic variation known for any pachycephalosaur, and thus is ideally suited to serve as a model for frontoparietal dome growth [10]. Here we perform the first analysis of frontoparietal allometry in *Stegoceras validum* utilizing morphological landmarks, and use this to further test the hypothesis that the flat-headed specimens are part of a continuous growth series in which cranial doming is tightly correlated with size change concomitant with ontogenetic growth.

### Frontoparietal Bone Histology

Osteohistology, the study of bone microstructure, is used to study the ontogeny of extant and extinct animals [24]–[34]. However, these studies have largely been confined to the analysis of limb bones, which is not feasible in pachycephalosaurs due to the general lack of postcranial remains. Osteohistology of pachycephalosaurs has been studied exclusively in cranial bone, which is dermal in origin and formed by intramembranous ossification, unlike limb bones which are formed by endochondral ossification [35]. Despite differences in the bone formation process, the osteohistology and bone growth in cranial bones should be similar to that of long bones because both types of bone contain the same materials and both undergo similar environmental variability [35]. As a result, differences in tissue types and structures observable between juvenile and adult limb bones should also be observable between juvenile and adult cranial bone [36].

Cranial histology of pachycephalosaurs has previously been examined by Brown and Schlaikjer [12], Goodwin et al. [15], Goodwin and Horner [37], and Horner and Goodwin [16]. The first two studies described the cranial histology of a single specimen. The study of Goodwin and Horner [37] used histological sections of frontoparietal domes from a multi-taxic size series to test functional hypotheses of this unusual structure. Horner and Goodwin [16] used histology and computed tomography of frontoparietals and cranial ornamentation as support for the hypothesized synonymy of *Dracorex*, *Stygimoloch*, and *Pachycephalosaurus*, but were unable to analyze the histology of all three taxa. While these studies were purely qualitative, they suggested that the relative vascularity of the dome decreased with ontogenetic age [37]. A preliminary study by Goodwin et al. [38] suggested the use of high-resolution computed tomography (HRCT) as a non-destructive method for examining bone histology. That study qualitatively compared cranial histology from HRCT scans of AMNH 5450 (the holotype of *Ornatotholus browni*) and TMP 84.5.1 (a small domed specimen of *Stegoceras validum*) and found both to be similar, a result that they suggested supports the synonymy of *Ornatotholus* and *Stegoceras* [38].

Following the work of Goodwin and Horner [37] and Goodwin et al. [38], we use HRCT scans from three specimens in our growth series of *Stegoceras validum* to quantitatively test the hypothesis that cranial vascularity decreases with ontogenetic age in pachycephalosaurs. Additionally, we provide qualitative observations on bone histology in these specimens. The three specimens HRCT scanned for this study are AMNH 5450, the holotype of *Ornatotholus browni* and what is here and elsewhere [9], [10], [15] considered a juvenile *S. validum*; TMP 84.5.1, a relatively small specimen of *S. validum* and presumed juvenile or subadult; and ROM 53555 one of the largest known specimens of *S. validum* and a presumed adult, although it is unclear if this specimen is fully mature. Based on the observations of Goodwin and Horner [37], we predict that flat-headed juvenile specimens will have a higher relative vascularity than fully domed specimens, and that vascular space will decrease with size through ontogeny.

### Institutional Abbreviations

**AMNH**, American Museum of Natural History, New York, New York, USA; **CMN**, Canadian Museum of Nature, Ottawa, Ontario, Canada; **MOR**, Museum of the Rockies, Bozeman, Montana, USA; **ROM**, Royal Ontario Museum, Toronto, Ontario, Canada; **TMP**, Royal Tyrrell Museum of Palaeontology, Drumheller, Alberta, Canada; **UALVP**, University of Alberta Laboratory of Vertebrate Paleontology, Edmonton, Alberta, Canada; **UCMP**, University of California Museum of Paleontology, Berkeley, California, USA; **UCMZ(VP)**, University of Calgary Museum of Zoology (Vertebrate Paleontology Collection), Calgary, Alberta, Canada; **Z**. **PAL**, Palaeozoological Institute, Warsaw, Poland.

## Materials and Methods

**Figure 1:**
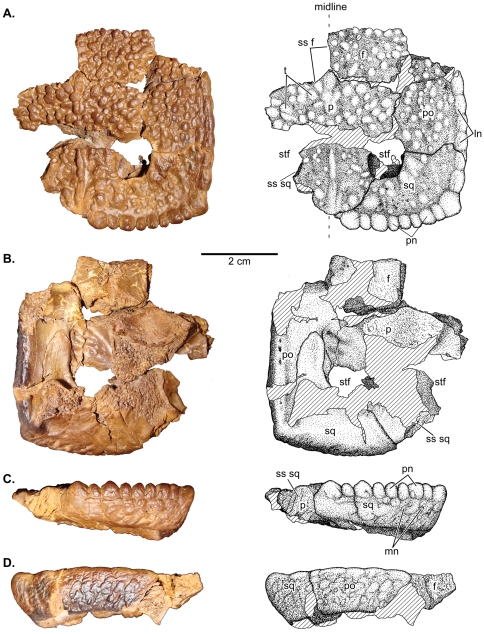
UCMZ(VP) 2008.001, a flat-headed juvenile *Stegoceras validum.* **A**, dorsal, **B**,ventral; **C**, posterior; and **D**, lateral views. Abbreviations: **cf**, cerebellar fossa; **f**, frontal; **ln**, lateral nodes; **o**, orbit; **p**, parietal; **pn**, posterior nodes; **po**, postorbital; **sq**, squamosal; **ss**, sutural surface for; **stf**, supratemporal fenestra.

**Figure 2:**
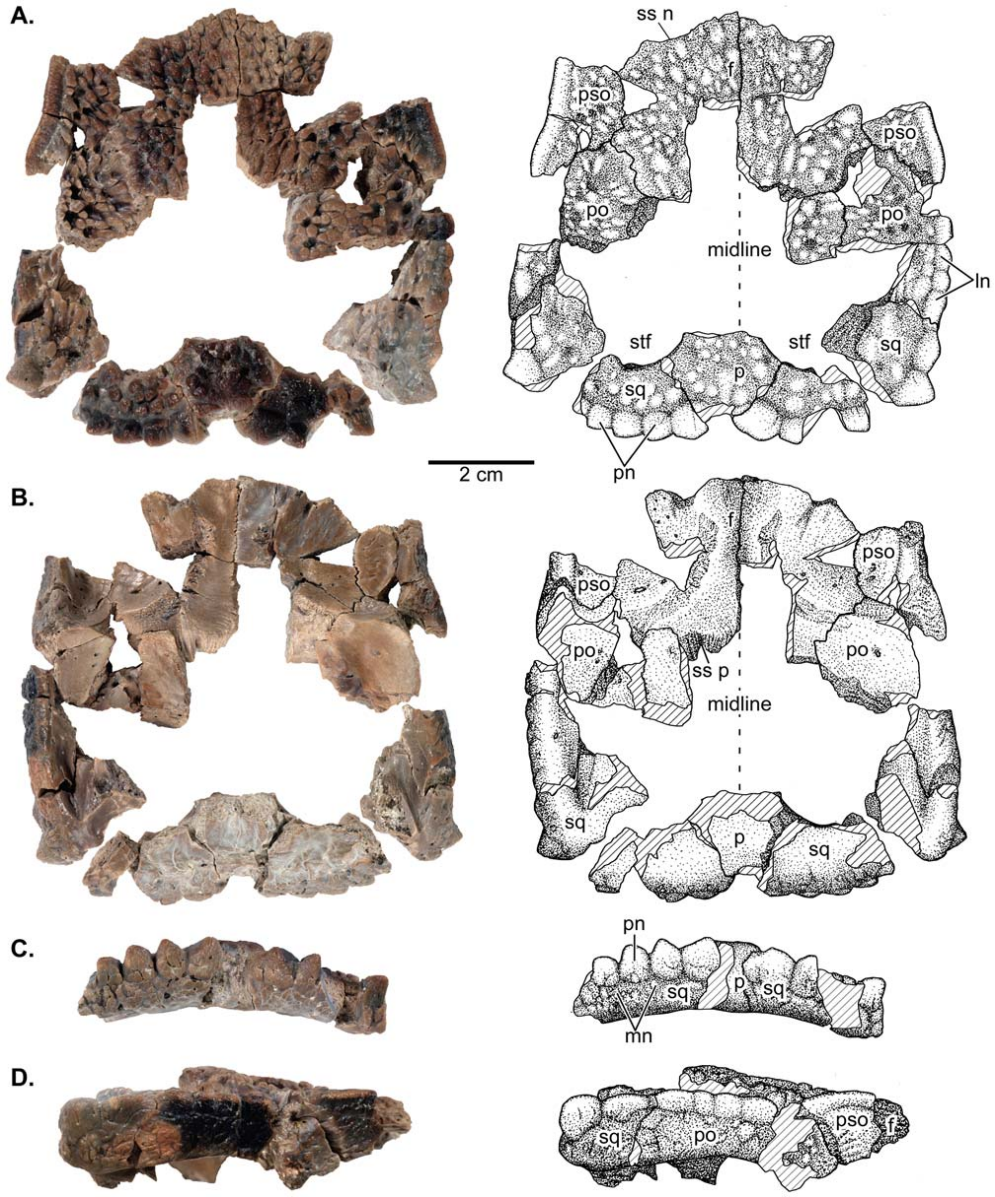
UALVP 49531, a flat-headed juvenile *Stegoceras validum.* **A**, dorsal; **B**,ventral; posterior, and lateral views. Abbreviations as in Figure 1.

Two new flat-headed *Stegoceras* specimens, UCMZ(VP) 2008.001 (Fig. 1) and UALVP 49531 (Fig. 2), are described based on first-hand examination. Comparisons were made to all other pachycephalosaur taxa, for which the vast majority of specimens were also examined first-hand, with the notable exception of *Goyocephale*, which was only studied through the literature. A complete list of *Stegoceras* material examined as part of this study is available in Table S1.

### Frontoparietal Allometry

**Figure 3:**
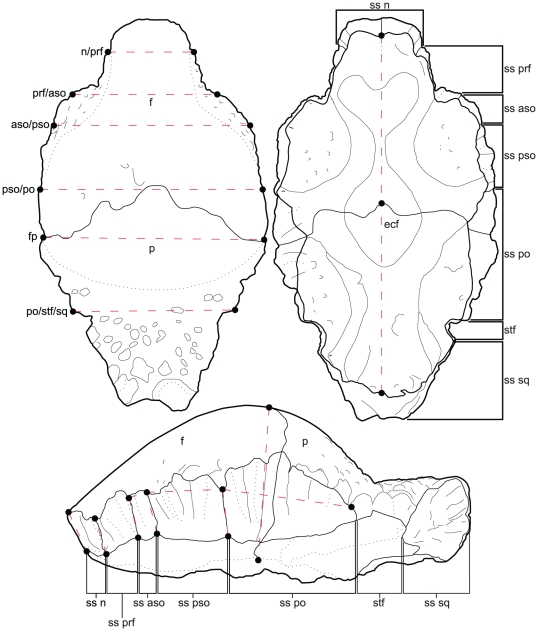
Illustration of the 18 linear measurements taken between 26 homologous landmarks. Abbreviations: **aso**, anterior supraorbital; **f**, frontal; **fnb**, frontonasal boss; **n**, nasal; **p**, parietal; **prf**, prefrontal; **po**, postorbital; **pso**, posterior supraorbital; **sq**, squamosal; **ss**, sutural surface for; **stf**, supratemporal fenestrae.

**Figure 4:**
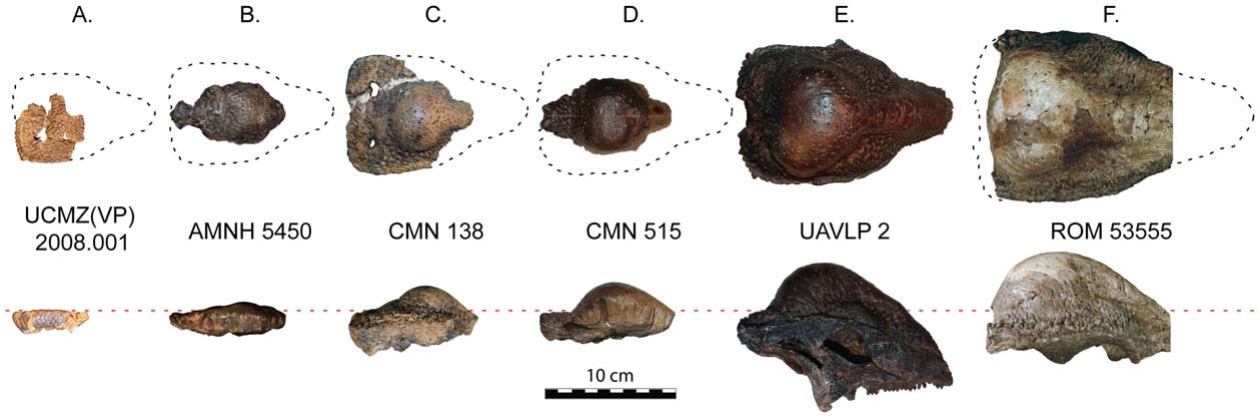
Growth series of *Stegoceras validum* in dorsal (top) and lateral (bottom) views. This series depicts the transition from a flat-headed to domed frontoparietal morphology that occurred through ontogeny in this taxon. **A**, UCMZ(VP) 2008.001 **B**, AMNH 5450 (holotype of *Ornatotholus browni*); **C**, CMN 138; **D**, CMN 515 (lectotype of *Stegoceras validum*); **E**, UALVP 2; **F**, ROM 53555. Portions of ROM 53555 are reconstructed including the nasals (cropped) and the lateral margins. Reconstructed portions are visible as dark grey areas in the CT images (Fig. 9).

Frontoparietal allometry was analyzed using landmark-based frontoparietal heights, lengths, and widths, for a total of 18 measurements (Fig. 3). All measurements were taken between homologous morphological landmarks (based, in part, on those identified by Goodwin, [23]) recorded at sutural contacts. A description of the individual measurement indices is found in Text S1. Measurements were taken, where preserved, from 40 specimens of *Stegoceras validum* (including *Ornatotholus browni*, AMNH 5450) representative of the hypothesized growth series (Fig. 4, Table S1). All of the specimens analyzed in this study are from the upper Belly River Group of southern Alberta, and have primarily been surface collected with little detailed locality data. All specimens with reasonably precise locality data occur in the Dinosaur Park Formation. Although none of the specimens can be positively sourced from the Oldman Formation (sensu Eberth and Hamblin [39]), historically collected specimens could be derived from either the Oldman or Dinosaur Park Formations. The majority of specimens have been previously referred to *S. validum* on the basis of their distinct morphology [40], [41] with the arguable exception of the *O. browni* holotype (AMNH 5450). The sample purposely excludes specimens reported from Montana or New Mexico [23], [42] to minimize the chances of sampling multiple taxa. Furthermore, specimens of *Stegoceras* from New Mexico have recently been referred to a new species [43]. Specimens and their measurements are listed in Table S1.

Measurements were log-transformed to fit the linear allometric growth function [44]. This has the additional benefit of increasing the normality of the data [45]. Regressions were calculated using reduced major axis regression (RMA, also known as standard major axis regression) implemented in the ‘lmodel2’ software package [46], [47] for the statistics program R. RMA has been suggested to be the most appropriate method for tests of allometry [48], [49]. Statistical significance of the allometric pattern was determined based on the 95% confidence intervals of the allometric coefficient (e.g., if the confidence interval encompasses one the slope is statistically isometric). Due to the varying completeness of specimens, four different standard (*x*) variables were used: frontoparietal length (L:fp), frontoparietal width (W:fp), parietal length (L:p), and frontal length (L:f). Frontoparietal length is perhaps the most intuitive index of size, however only ten specimens have complete frontoparietals. We also used parietal length (L:p) and frontoparietal width (W:fp) as standard variables for comparisons in order to include the flat-headed specimens and maximize sample size (and thus statistical power). In these cases, it was possible to include isolated frontals and/or parietals in the bivariate analyses.

Dodson [50] found that intraspecific allometries in a growth series of extant *Alligator mississippiensis* showed very high correlation coefficients, and thus we consider a high correlation coefficient as support for the hypothesis that the specimens of *Stegoceras validum* represent an ontogenetic series of a single species. In order to further test the hypothesis that flat-headed specimens (including the *Ornatotholus browni* holotype, AMNH 5450) are juveniles of domed specimens, as well as to assess the effect of the flat-headed specimens on the regressions, we performed a series of comparisons in which flat-headed specimens were excluded. This was performed in two stages. First, all specimens less thick than TMP 84.5.1, the smallest domed specimen that is unequivocally *S. validum* (based on the presence of an articulated squamosal), were excluded. This removed all flat-headed specimens, some partially domed frontals, and the holotype of *O. browni* (AMNH 5450). At the second stage, we returned the three partially domed frontals (TMP 78.19.04, TMP 86.71.2, and TMP 2002.12.57) to the sample and reran the regressions.

#### Measurement Abbreviations

**H:n/n**, height of the sutural surface at the contact of the nasals; **H:n/prf**, height of the sutural surface at the contact of the nasal and prefrontal; **H:prf/aso**, height of the sutural surface at the contact of the prefrontal and anterior supraorbital; **H:aso/pso**, height of the sutural surface at the contact of the anterior supraorbital and posterior supraorbital; **H:pso/po**, height of the sutural surface at the contact of the posterior supraorbital and postorbital; **T:fp**, thickness of the frontoparietal; **L:aso**, length of the supraorbital suture; **L:pso**, length of the posterior supraorbital suture; **L:po**, length of the postorbital suture; **L:f**, length of the frontal; **L:p**, length of the parietal; **L:fp**, length of the frontoparietal; **W:n/prf**, width between the nasal/prefrontal sutural contacts; **W:prf/aso**, width between the aso/pso supraorbital sutural contacts; **W:aso/pso**, width between anterior and posterior supraorbital sutural contacts; **W:pso/po**, width between the posterior supraorbital and postorbital sutural contacts; **W:fp**, width of frontoparietal at the contact between the frontal and parietal; **W:po/stf/sq**, width between the contacts of postorbital suture and the supratemporal fenestrae or the squamosal suture if fenestrae are closed.

### Frontoparietal Bone Histology

HRCT scans were chosen as a non-destructive alternative to traditional thin sectioning for examining gross differences in bone histology [38]. Beyond the benefit of being non-destructive, HRCT slices are akin to serial sections and allow the gross histology to be mapped throughout the entire specimen within the context of a three-dimensional virtual model that is easily manipulated with a visualization program. Three specimens representing three stages in the ontogenetic growth series of *Stegoceras validum* were HRCT scanned in their entirety for this study (AMNH 5450, TMP 84.5.1, ROM 53555). Scans were performed by the University of Texas High-Resolution X-ray CT Facility (UTCT) and the analytical details of each scan are provided in Text S2. Digital data are archived at the Royal Ontario Museum. Histological comparisons were made between the scans of the three specimens including an assessment of the Zones proposed by Goodwin and Horner [37]. Additionally, we were given access to CT scans of UALVP 2. This specimen is similar to ROM 53555 in size and thus we would expect similar histological results for this UAVLP 2. Unfortunately, UAVLP 2 was not scanned in the same orientation, nor with the same level of resolution (due to the larger size of a complete skull), as the three specimens scanned for this study, and thus is not directly comparable. Because of this we only briefly report our results for this specimen.

#### Relative Vascularity

Goodwin and Horner [37] equate vascularity of pachycephalosaur domes directly with void space within the fossil bone. However, vascularity cannot be measured directly in fossil specimens because blood vessels only occupy a small portion of the bony canals that host them, together with other tissues [32]. Although an unequivocal functional relationship between size of the bony canals, or extent of void space, in bone and vascularization has not yet been well established [32], void space in the CT scans was chosen as a proxy for vascularity, as these spaces were certainly occupied by blood vessels in life, and Goodwin and Horner's [37] histological model of pachycephalosaur cranial ontogeny is based on the relative proportion of void space in the dome. Additionally, because we are comparing relative changes, the assumptions of this method will have less of an impact as long as the correlation is consistent between the specimens.

Void space was calculated using transverse sections from the CT data. A single section was chosen at the boundary of the posterior supraorbital and postorbital sutures on the frontal from each of the three specimens. This ensured that each section was homologous and thus directly comparable to the other sections. Void space was calculated using a threshold-based technique implemented in ImageJ [51], [52], which creates a binary image by specifying a grayscale value under which all pixels are made black and over which all pixels are made white. There are numerous different methods for determining the threshold value and while some have been found to perform better than others, the best method is still highly dependent on the images being analyzed [53]. We chose the Huang method [54], [55], which minimizes fuzziness in the 2-D grayscale histogram, based on a survey of different thresholding techniques, as the method that best approximated what we interpreted as the void/canal space in the scan.

To properly compare relative void spaces, a region of interest was specified by a rectangle of the same relative proportions drawn onto each thresholded image at the base of the braincase immediately to the left of the interfrontal suture and extended so that the top left corner met the edge of the frontal. This technique ensured that homologous areas were compared despite differences in the shapes of the frontals. This step was also used to eliminate any potential artifacts, such as the effects of beam hardening which can result in the edge of the specimen being recognized as void space. Thresholded images were cropped to this region of interest and relative void space was calculated using the voxel counter plug-in for ImageJ [51], [52].

## Results

### Systematic Paleontology

Dinosauria Owen 1842 [56]

Ornithischia Seeley 1887 [57]

Pachycephalosauria Maryańska and Osmólska 1974 [58]

Pachycephalosauridae Sternberg 1945 [3]

*Stegoceras* Lambe 1902 [59]

*Stegoceras validum* Lambe 1902 [59]

#### Lectotype

CMN 515, nearly complete frontoparietal dome.

#### Type Locality and Horizon

East side of the Red Deer River below the mouth of Berry Creek, Alberta; Dinosaur Park Formation (Campanian).

#### Synonymy

*Stegoceras browni* Wall and Galton 1979 [13]; *Ornatotholus browni* Galton and Sues 1983 [14], following Sullivan 2003 [9].

#### Referred Specimens

A complete list of referred specimens is included in Table S1. Newly referred specimens important to the descriptive section of this study include: UCMZ(VP) 2008.001, partial right frontal, fused parietals, right squamosal, and right postorbital (Fig. 1); UCMZ 2008.002, isolated flat parietal; UALVP 49531, partial/fragmentary squamosals, parietals, frontals, postorbitals, supraorbitals (Fig. 2).

#### Locality and Horizon

All specimens were recovered from the Belly River Group of Alberta, Canada, with the majority of specimens from the Dinosaur Park Formation in the area of Dinosaur Provincial Park. Detailed locality data for individual specimens is available to qualified researchers from their respective institutions. Locality information for new specimens described in this paper is as follows: UCMZ(VP) 2008.001, Steveville Railway Grade, Dinosaur Park Formation, 37 m above the contact with the Oldman Formation, Alberta; UALVP 49531, Steveville area, Dinosaur Park Formation, approximately 40 m above the contact with the Oldman Formation, Dinosaur Provincial Park, Alberta.

#### Emended Diagnosis

A domed pachycephalosaur that differs from all other pachycephalosaurs in having a parietosquamosal shelf with ornamentation consisting of numerous minute tubercles on lateral and posterior sides of squamosals, a prominent dorsal row of five to eight dorsally projecting primary nodes on each side of parietosquamosal bar, and a row of small, keel-shaped nodes on lateral margin of squamosal. Incorporation of peripheral elements, particularly supraorbitals, into dome is not developed to the extent found in *Prenocephale*, *Sphaerotholus*, and *Pachycephalosaurus*. Differs from all other pachycephalosaurs, where known, in absence of nasal ornamentation, greatly reduced diastema in upper tooth row, and pubic peduncle of the ilium mediolaterally compressed and plate-like (modified, in part, from Sullivan [9]).

#### Comments

This study shows that a number of features, including some listed as diagnostic by Sullivan [9], are variable in *Stegoceras validum*. This includes the extent of doming, which increases ontogenetically; the degree of closure of the supratemporal fenestrae, which shows a large amount of individual variation in addition to the tendency to close ontogenetically; and the prominence of the parietosquamosal shelf, which decreases with ontogeny. Variation in these, and other, features is discussed further in the following sections.

### Description

Here we describe two recently collected flat-headed specimens of *Stegoceras validum* UCMZ(VP) 2008.001 and UALVP 49531, which are the first flat-headed specimens from the Dinosaur Park Formation with associated squamosals. The ornamentation of the parietosquamosal bar is diagnostic in pachycephalosaurs and allows these specimens to be assigned to *S. validum*.

#### UCMZ(VP) 2008.001

UCMZ(VP) 2008.001 (Fig. 1) consists of a complete parietal, right squamosal, right postorbital, and incomplete right frontal preserved in articulation. The dorsal surfaces of the bones form a completely flat surface except for the small tubercle-like projections, which form the nodular surface texture present in many pachycephalosaurs. This surface texture is identical to that covering the uninflated portions of the cranial domes of *Stegoceras* [10] and is consistent with the surface texture thought to typify *Ornatotholus browni* [14].

Only the posterior portion of the right frontal of UCMZ(VP) 2008.001 is preserved; the sutural surfaces for the nasal, prefrontal, anterior supraorbital, and a portion of the posterior supraorbital bones are missing. The preserved width of the right frontal is 20 mm.

The medial wall of the frontal is relatively straight anteroposteriorly and bears the striated sutural surface for the left frontal. The anterior portion of the lateral wall is also straight anteroposteriorly and preserves a small portion of the sutural surface for the posterior supraorbital. Most of the lateral margin is damaged posteriorly. However, a portion of the ventral surface of the frontal articulates with the postorbital. The posterior region of the frontal contacts the parietal in a pronounced, slightly interdigitated butt joint. The ventral surface of the frontal preserves the anterior portion of the cerebral fossa medially and a small portion of the roof of the right orbital cavity laterally. The rugose sutural surface for the orbitosphenoid separates the cerebral and orbital fossae. The surfaces of cerebral and orbital fossae are smooth and slightly concave. The orbital fossa is pierced by several prominent foramina.

The parietal is nearly complete, with only a small band of bone missing (mainly dorsally) between the anterior margins of the supratemporal fenestrae. The parietal is 33.6 mm wide at the frontoparietal suture and 37.4 mm in length along the midline. The open supratemporal fenestrae are prominent. The maximum diameter of the supratemporal fenestra is 11.6 mm, and the minimum distance between the supratemporal fenestrae is 17.3 mm.

The parietal contacts the frontals anteriorly along a relatively straight transverse suture. Medially, a distinctly pointed interfrontal process of the parietal projects between the posteromedial ends of the frontals. Most of the ventral portion of the sutural surface is not preserved, but the anteroventral region of the bone deepens laterally such that the height of the frontoparietal suture (and participating bones) increases laterally. The anterolateral wall of the parietal has an extensive parasagittal contact with the postorbital. The lateral wall forms the anterior and medial margins of the large circular supratemporal fenestrae. On the right side, the finished surface of the margin of the supratemporal fenestrae is missing; however the concavity of the surface is evident. On the left side, the anterior margin of the supratemporal fenestrae is missing.

Posterior to the supratemporal fenestrae, the posteromedial process of the parietal is triangular, in dorsal view, and tapers to a small slip between the squamosals. The squamosal is articulated with the parietal on the right side, whereas the complete sutural surface is exposed on the left side where the left squamosal is missing. The posterior-most section of the parietal is damaged, but it is clear that the parietal was very narrow between the squamosals in posterior view and may have been excluded entirely from the posterior surface of the parietosquamosal bar. The posterior exposure of the posteromedial extension of the parietal is highly variable in *Stegoceras validum* and may be widely exposed as in UALVP 2, or nearly excluded posteriorly as in the lectotype, CMN 515 (see also Sullivan [9]).

The ventral surface of the parietal is formed of the posterior half of the cerebral fossa anteromedially, whereas the posterior region that supports the supraoccipital consists mainly of broken bone surface. The lateral portion of the ventral surface of the parietal contains portions of the temporal chambers. The medial wall of the temporal chamber is highly concave, and the relatively flat dorsal surface is pierced by several foramina.

Only the right squamosal is preserved, which is complete except for its ventral projections. The overall morphology of the squamosal is typical of *Stegoceras validum* (e.g., CMN 138, CMN 8816, TMP 84.5.1, UALVP 2), where it forms the posterolateral margin of the supratemporal fenestra anteromedially, and contributes to the ornamented parietosquamosal and squamosopostorbital bars posterolaterally. The squamosal is 32.3 mm in mediolateral width and 17.8 mm in anteroposterior length.

Anteriorly, the articulated squamosal contacts the right postorbital. The sutural surface is sinuous in dorsal view, a condition also present in other specimens of *Stegoceras validum* (e.g., CMN 138). The squamosal has an irregular, nodal surface texture on its lateral surface that continues anteriorly onto the postorbital. The dorsolateral edge has two thin, low, anteroposteriorly long nodes that contribute to a row of five similarly shaped nodes that make up the lateral (squamosopostorbital) node row (excluding the large pyramidal vertex node at the junction between the posterior and lateral node rows). These nodes are morphologically distinct from the posterior (parietosquamosal) nodes.

The medial wall of the squamosal forms the lateral and posterior margins of the supratemporal fenestra. The squamosal contributes to nearly half the margin of the fenestra. The squamosal contacts the parietal to form the parietosquamosal bar along the caudal margin of the skull. In posterior view, the squamosal almost reaches the midline posterior to the parietal, but it is unclear whether it contacts the opposite squamosal due to incomplete preservation. The posterior bar of the squamosal increases in dorsoventral height laterally, as in other specimens of *Stegoceras validum* [10]. The posterodorsal margin of the squamosal has eight dome-shaped nodes that form the posterior (parietosquamosal) node row. This is slightly greater than expected for *S. validum*, but the number of squamosal nodes forming the posterior node row is variable in this taxon, ranging from five to at least seven (see Sullivan [9]). In UCMZ(VP) 2008.001, the restriction of the parietal between the squamosals may account for the increased number of nodes. The nodes are variable in width, with the medial-most being the largest, but are nearly all identical in height. Many small, irregularly-sized nodes (minute nodes) that do not form distinguishable rows or clusters, a condition typical of *S. validum* (e.g., CMN 138, CMN 8816, TMP 84.5.1, UALVP 2), are present below the posterior node row. At the corner of the squamosal, where the lateral and posterior borders meet, a pyramidal node (the vertex node), larger than the other nodes, connects the posterior and lateral node rows.

The ventral surface of the squamosal is smooth (anodal) where preserved. Broken regions demarcate the bases of the occipital plate and the quadrate processes, which are missing. Medially, the postorbital process of the squamosal extends anteriorly and underlies the ventral surface of the postorbital in a tongue-like projection, a condition typical in *Stegoceras validum* (e.g., CMN 138) and pachycephalosaurs in general.

The right postorbital is nearly complete, with only a small portion missing on the anterolateral surface. The rugose anterior surface marks the contact of the posterior supraorbital. The postorbital contacts the parietal medially and the squamosal posteriorly and is 24.8 mm long anteroposteriorly and 18.2 mm wide mediolaterally.

In dorsal view, the suture for the posterior supraorbital is angled posterolaterally, and extends further posteriorly immediately before its lateral margin. This pattern is observed in other specimens of *Stegoceras validum* (e.g., CMN 138). The medial wall of the postorbital is oriented posteromedially. The anterior-most portion of the medial wall would have contacted the frontal, but is not preserved. Posterior to the medial wall, the postorbital contacts the parietal. The posterior-most part of the medial wall forms a small section of the anterolateral border of the supratemporal fenestrae. In dorsal view, the lateral wall of the postorbital is relatively straight. The dorsolateral edge contains four low nodes identical to, and continuous with, those on the dorsolateral margin of the squamosal. In lateral view, the postorbital wall is relatively flat with a subtle surface texture that is much less distinct than on the dorsal surface. Posteriorly, the postorbital contacts the squamosal in a complex, interlapping joint.

#### UALVP 49531

UALVP 49531 consists of a fragmented frontoparietal and incomplete peripheral skull elements including the frontals, parietal, squamosals, postorbitals, and posterior supraorbitals (Fig. 2). Although many of the bones are incomplete, the proportions of the skull roof can be accurately estimated due to the preserved articulations. The dorsal surface texture of the bones and the shape of the flat frontal and parietal are similar to UCMZ(VP) 2008.001.

The preserved portions of the squamosals and posteromedial extension of the parietal indicate open supratemporal fenestrae that are proportionately larger than in UCMZ(VP) 2008.001. Both squamosals are incomplete, but a portion of the primary posterior node row is preserved. The posterior node row is incomplete on both sides preventing determination of the total number of nodes. Four nodes are preserved on the partial left squamosal and we estimate that at least one to two additional nodes are missing. The primary posterior squamosal nodes of UALVP 49531 show a marked difference in morphology from UCMZ(VP) 2008.001. The preserved nodes in UALVP 49531 are larger, subtriangular, and more widely spaced than in UCMZ(VP) 2008.001, but closely resemble the nodes of UALVP 2. Both domed and triangular node morphologies are present and within the range of variation of *Stegoceras validum* specimens from Dinosaur Provincial Park (e.g., UALVP 2, CMN 138, TMP 84.5.1). The morphology and sutural contracts of the postorbital are identical to UCMZ(VP) 2008.001. The partial left squamosal and partial left postorbital preserve the lateral node row with a large vertex node that would have connected the lateral and posterior node rows. The five preserved lateral nodes are similar in shape, although they are slightly larger and more distinct than those in UCMZ(VP) 2008.001.

The left posterior supraorbital is nearly complete, missing only a small portion medially where it articulates with the postorbital. The dorsal surface texture of the posterior supraorbital is tuberculate as in the other bones. The anterior sutural surface for articulation with the anterior supraorbital is relatively straight except where it extends ventrally and slightly posteriorly at the lateral edge. The lateral edge forms the postorbital-supraorbital bar, which is devoid of any nodes and slightly convex anteroposteriorly. Ventral to the bar, the lateral wall is slightly concave mediolaterally, but otherwise flat with a surface texture similar to the minute nodes present on the posterior wall of the squamosal, although somewhat less distinct. The posterior sutural surface for articulation with the postorbital is narrow and angles anteriorly. Medially, the supraorbital contacts the frontal along a straight suture until it is excluded from contact by the postorbital. At this point the lateral portion of the posterior supraorbital continues posteriorly along the anterolateral edge of the postorbital. In ventral view, the medial portion of the posterior supraorbital forms the posterolateral portion of the slightly concave roof of the orbital cavity. In this same view, the lateral portion forms a convex ridge that extends ventrally and would have formed the dorsal margin of the orbit if more completely preserved.

### Taxonomic Assessment

The specimens UCMZ(VP) 2008.001 and UALVP 49531 consist of flat frontals, parietals, and the diagnostic squamosal and postorbital bones. The shape and ornamentation of these elements are within the range of variation of *Stegoceras validum* (e.g., UALVP 2, CMN 138, TMP 84.5.1). The squamosal of UCMZ(VP) 2008.001 has one additional node (eight) on its posterior edge than previously found in *S. validum*, but the number of nodes is variable in this taxon (e.g., CMN 138, TMP 84.5.1, UALVP 2). The shape and distribution of these nodes, including the distribution of minute nodes on the posterior edge of the squamosal, is virtually identical to *S. validum*. The squamosal of UALVP 49531, with an estimated six to seven nodes in the primary node row, is within the range of variation observed for *S. validum* (e.g., UALVP 2, CMN 138, TMP 84.5.1). Additionally, the shape and surface texture of the squamosal and postorbital in both specimens is also observed in small specimens of *S. validum* (e.g., CMN 138, TMP 84.5.1).

Unfortunately, the small sample size of pachycephalosaurs with precise stratigraphic data makes it difficult to assess whether any of the variation in parietosquamosal ornamentation is correlated with stratigraphic position within the host unit, as in other better documented orntihischians from Dinosaur Provincial Park [40]. The flat-headed specimens UCMZ(VP) 2008.001 and UALVP 49531 are from 37 m and 40 m above the Oldman-Dinosaur Park Formation contact, respectively, whereas UALVP 2 is from approximately 22 m above this boundary.

The flattened frontals and parietals described here closely resemble those previously referred to *Ornatotholus browni* (e.g., TMP 78.19.4, Galton and Sues [14]; UCMP 130295, Goodwin [23]), which suggests they could be referred to this taxon, if it was valid (see Discussion below). However, the diagnostic nature of the squamosal of *Stegoceras validum* (e.g., Sullivan [9]) indicates that UCMZ(VP) 2008.001 and UALVP 49531 can be referred to *S. validum*. In fact, the distinct nature of the these squamosals and their associated ornamentation precludes us from referring UCMZ(VP) 2008.001 and UALVP 49531 to any other known species. Given the diagnostic and consistent nature of the morphology of squamosal ornamentation, and that these specimens occur in the same host formation and geographic area as known *S. validum* specimens, UCMZ(VP) 2008.001 and UALVP 49531 are identified here as juvenile individuals of *S. validum.* Other flat frontals and parietals previously referred to *O. browni* (e.g., TMP 78.19.4) are suggestive of *S. validum* based on their similarity with UCMZ(VP) 2008.001 and are also referred to this taxon. This would corroborate the hypothesis of ontogenetic change from flat-headed to domed morphology in *S. validum* proposed by Goodwin et al. [15], Williamson and Carr [10], and Sullivan [9]. The allometric and histological analyses performed in the following sections are used to further test this hypothesis and to quantitatively describe ontogenetic change in *Stegoceras*.

### Frontoparietal Allometry

**Figure 5:**
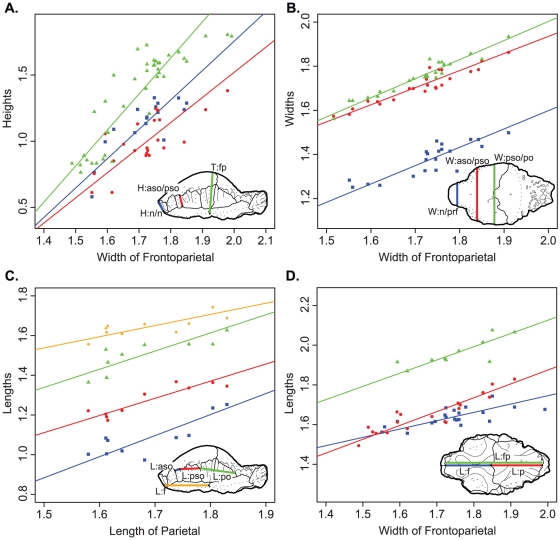
Bivariate logarithmic plots with RMA regression lines for selected variables. **A**, heights of frontoparietal vs width across frontoparietal suture. **Blue**, height of nasal; **red**, height of frontoparietal at the contact of the anterior and posterior supraorbitals; **green**, thickness of frontoparietal. **B**, widths of frontoparietal vs width across frontoparietal suture. **Blue**, width of frontonasal boss; **red**, width across the supraorbital lobes; **green**, width across the frontoparietal at the contact of the posterior supraorbital and postorbital sutures. **C**, lengths of frontoparietal vs width across frontoparietal suture. **Blue**, length of frontal; **red**, length of parietal; **green**, length of frontoparietal. **D**, lengths of frontoparietal vs length of parietal. **Blue**, length of anterior supraorbital suture; **red**, length of posterior supraorbital suture; **green**, length of postorbital suture; **orange**, length of frontal. Measurements are log (mm).

**Figure 6:**
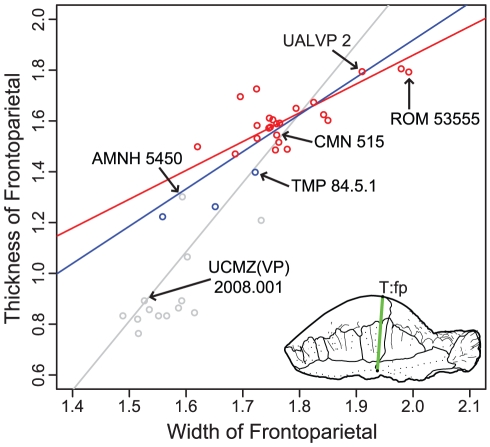
Bivariate logarithmic plots with RMA regression lines for frontoparietal thickness vs. width. **Red**, only specimens more domed (thicker) than TMP 84.5.1; **blue**, includes the three partially domed frontals; and **grey**, with all specimens included. Measurements are log (mm).

Results of the bivariate comparisons are listed in Tables 2, 3, 4, 5, 6, 7, 8, 9, 10, 11, 12, 13, 14, 15, 16, 17, and graphs of select indices are presented in Figures 5 and 6. Regressions using frontoparietal width (W:fp) as the standard variable have the highest sample size, and thus the greatest statistical power. Comparisons using frontoparietal length (L:fp) and parietal length (L:p) have lower sample sizes that may hinder the ability to detect positive and negative allometry statistically. The correlation coefficients using W:fp, L:p, and L:fp as standard variables, are generally fairly high (most are >0.7, see Tables 2, 3, 4, 5, 6, 7, 8, 9, 10, 11), but they are not as high as those reported by Dodson for *Alligator* [50]. Dodson [50] noted that the high correlation coefficients of his results were partially due to the large size range of his *Alligator* sample; the size range of *Stegoceras* specimens is not nearly as large, and thus we would expect to find somewhat lower correlation coefficients even in an intraspecific ontogenetic series. Comparisons using frontal length (L:f) as the standard variable, while having relatively high sample sizes, tended to have much lower correlation coefficients (0.35–0.56). This suggests that L:f is not ideal as a standard variable, most likely due to a high amount of individual variation in frontal length.

**Table 2:** Allometric regression of *Stegoceras validum* frontoparietal heights against frontoparietal width.

Frontoparietal Width							
	N	R	Slope	CI	Intercept	CI	Allometry
H:n/n	17	0.81	2.22	1.62–3.05	−2.68	−4.12–−1.65	+
H:n/prf	16	0.74	2.14	1.47–3.13	−2.59	−4.27–−1.44	+
H:prf/aso	26	0.78	2.22	1.71–2.88	−2.78	−3.91–−1.91	+
H:aso/pso	22	0.75	1.9	1.40–2.58	−2.27	−3.44–−1.41	+
H:pso/po	24	0.82	1.81	1.41–2.33	−1.98	−2.87–−1.29	+
T:fp	39	0.87	2.72	2.32–3.20	−3.27	−4.08–−2.58	+

Abbreviations: **CI**, 95% confidence interval; **iso**, isometry; **+**, positive allometry; **−**, negative allometry; **H:n/n**, height of the sutural surface at the contact of the nasals; **H:n/prf**, height of the sutural surface at the contact of the nasal and prefrontal; **H:prf/aso**, height of the sutural surface at the contact of the prefrontal and anterior supraorbital; **H:aso/pso**, height of the sutural surface at the contact of the anterior supraorbital and posterior supraorbital; **H: pso/po**, height of the sutural surface at the contact of the posterior supraorbital and postorbital; **T:fp**, thickness of the frontoparietal.

**Table 3:** Allometric regression of *Stegoceras validum* frontoparietal heights against frontoparietal length.

Frontoparietal Length							
	N	R	Slope	CI	Intercept	CI	Allometry
H:n/n	9	0.72	1.75	0.98–3.14	−2.25	−4.95–−0.74	iso
H:n/prf	7	0.76	1.48	0.74–2.95	−1.8	−4.69–−0.36	iso
H:prf/aso	10	0.9	1.46	1.03–2.09	−1.75	−2.97–−0.89	+
H:aso/pso	8	0.87	2.07	1.28–3.35	−3.04	−5.51–−1.50	+
H:pso/po	10	0.72	1.38	0.80–2.36	−1.51	−3.44–−0.38	iso
T:fp	10	0.67	2.03	1.15–3.60	−2.44	−5.51–−0.71	+

Abbreviations as in Table 2.

**Table 4:** Allometric regression of *Stegoceras validum* frontoparietal heights against parietal length.

Parietal Length							
	N	R	Slope	CI	Intercept	CI	Allometry
H:n/n	9	0.83	1.38	0.85–2.23	−1.13	−2.56–−0.25	iso
H:n/prf	7	0.78	1.23	0.63–2.39	−0.96	−2.90–0.04	iso
H:prf/aso	11	0.66	2.23	1.30–3.83	−2.68	−5.37–−1.12	+
H:aso/pso	8	0.83	1.44	0.85–2.44	−1.4	−3.06–−0.42	iso
H:pso/po	10	0.84	1.06	0.69–1.63	−0.59	−1.55–0.03	iso
T:fp	19	0.77	3.81	2.76–5.26	−4.99	−7.38–−3.25	+

Abbreviations as in Table 2.

**Table 5:** Allometric regression of *Stegoceras validum* frontoparietal heights against frontal length.

Frontal Length							
	N	R	Slope	CI	Intercept	CI	Allometry
H:n/n	17	0.51	4.39	2.78–6.94	−6.04	−10.21–−3.40	+
H:n/prf	15	0.35	3.98	2.33–6.80	−5.43	−10.03–−2.74	+
H:prf/aso	19	0.51	4.05	2.64–6.22	−5.52	−9.06–−3.22	+
H:aso/pso	17	0.37	4.52	2.77–7.39	−6.35	−11.01–−3.50	+
H:pso/po	19	0.41	3.23	2.06–5.07	−4.09	−7.10–−2.17	+
T:fp	21	0.56	4.71	3.20–6.93	−6.18	−9.85–−3.70	+

Abbreviations as in Table 2.

**Table 6:** Allometric regression of *Stegoceras validum* frontoparietal widths against frontoparietal width.

Frontoparietal Width							
	N	R	Slope	CI	Intercept	CI	Allometry
W:n/prf	21	0.85	0.83	0.65–1.07	−0.06	−0.48–0.26	iso
W:prf/aso	25	0.84	0.77	0.61–0.97	0.27	−0.07–0.54	−
W:aso/pso	24	0.94	0.77	0.66–0.90	0.39	0.16–0.58	−
W:pso/po	24	0.96	0.87	0.77–0.98	0.27	0.08–0.44	−
W:po/stf/sq	15	0.93	0.87	0.70–1.08	0.11	−0.25–0.41	iso

Abbreviations: **CI**, 95% confidence interval; **iso**, isometry; **+**, positive allometry; **−**, negative allometry; **W:n/prf**, width between the nasal/prefrontal sutural contacts; **W:prf/aso**, width between the aso/pso supraorbital sutural contacts; **W: aso/pso**, width between anterior and posterior supraorbital sutural contacts; **W:pso/po**, width between the posterior supraorbital and postorbital sutural contacts; **W:po/stf/sq**, width between the contacts of postorbital suture and the supratemporal fenestrae or the squamosal suture if fenestrae are closed.

**Table 7:** Allometric regression of *Stegoceras validum* frontoparietal widths against frontoparietal length.

Frontoparietal Length							
	N	R	Slope	CI	Intercept	CI	Allometry
W:n/prf	9	0.7	1.24	0.68–2.26	−1.05	−3.05–0.05	iso
W:prf/aso	10	0.81	1.15	0.72–1.84	−0.65	−1.99–0.19	iso
W:aso/pso	10	0.84	1.11	0.72–1.70	−0.43	−1.60–0.32	iso
W:pso/po	9	0.89	1.22	0.82–1.80	−0.62	−1.77–0.16	iso
W:fp	10	0.8	1.5	0.93–2.41	−1.19	−2.98–−0.08	iso
W:po/stf/sq	8	0.93	1.29	0.91–1.82	−0.9	−1.96–−0.16	iso

Abbreviations as in Table 6.

**Table 8:** Allometric regression of *Stegoceras validum* frontoparietal widths against parietal length.

Parietal Length							
	N	R	Slope	CI	Intercept	CI	Allometry
W:n/prf	9	0.84	0.96	0.60–1.52	−0.22	−1.18–0.38	iso
W:prf/aso	11	0.93	0.91	0.69–1.19	0.08	−0.40–0.45	iso
W:aso/pso	10	0.93	0.85	0.64–1.14	0.3	−0.19–0.66	iso
W:pso/po	9	0.92	0.93	0.66–1.32	0.21	−0.45–0.67	iso
W:fp	19	0.91	1.43	1.16–1.77	−0.69	−1.24–−0.24	+
W:po/stf/sq	11	0.91	1.15	0.85–1.55	−0.32	−1.00–0.18	iso

Abbreviations as in Table 6.

**Table 9:** Allometric regression of *Stegoceras validum* frontoparietal lengths against frontoparietal width.

Frontoparietal Width							
	N	R	Slope	CI	Intercept	CI	Allometry
L:aso	26	0.79	1.18	0.92–1.52	−0.98	−1.57–−0.52	iso
L:pso	26	0.86	0.9	0.73–1.12	−0.31	−0.68–−0.01	iso
L:po	18	0.91	0.81	0.65–1.02	0.1	−0.26–0.38	iso
L:f	21	0.69	0.42	0.30–0.59	0.91	0.61–1.12	−
L:p	19	0.91	0.7	0.56–0.86	0.48	0.24–0.70	−
L:fp	10	0.8	0.67	0.41–1.07	0.79	0.08–1.23	iso

Abbreviations: **CI**, 95% confidence interval; **iso**, isometry; **+**, positive allometry; **−**, negative allometry; **L:aso**, length of the anterior supraorbital suture; **L:pso**, length of the posterior supraorbital suture; **L:po**, length of the postorbital suture; **L:f**, length of the frontal; **L:p**, length of the parietal; **L:fp**, length of the frontoparietal.

**Table 10:** Allometric regression of *Stegoceras validum* frontoparietal lengths against frontoparietal length.

Frontoparietal Length							
	N	R	Slope	CI	Intercept	CI	Allometry
L:aso	10	0.82	1.38	0.88–2.16	−1.62	−3.16–−0.64	iso
L:pso	10	0.69	1.12	0.64–1.97	−0.93	−2.59–0.02	iso
L:po	9	0.83	1.2	0.74–1.93	−0.85	−2.29–0.05	iso
L:f	10	0.93	0.73	0.54–0.99	0.21	−0.30–0.59	−
L:p	10	0.89	1.3	0.90–1.86	−0.86	−1.97–−0.087	iso

Abbreviations as in Table 9.

**Table 11:** Allometric regression of *Stegoceras validum* frontoparietal lengths against parietal length.

Parietal Length							
	N	R	Slope	CI	Intercept	CI	Allometry
L:aso	10	0.81	1.06	0.67–1.68	−0.71	−1.76–45955	iso
L:pso	10	0.91	0.86	0.62–1.21	−0.19	−0.77–0.23	iso
L:po	9	0.81	0.92	0.56–1.51	−0.03	−1.03–0.57	iso
L:f	10	0.76	0.56	0.34–0.93	0.69	0.07–1.07	−

Abbreviations as in Table 9.

**Table 12:** Allometric regression of *Stegoceras validum* frontoparietal heights against frontoparietal width excluding all specimens less domed (thick) than TMP 84.5.1.

Frontoparietal Width							
	N	R	Slope	CI	Intercept	CI	Allometry
H:n/n	14	0.51	1.58	0.94–2.65	−1.56	−3.43–−0.44	iso
H:n/prf	12	0.36	2.17	1.17–4.01	−2.64	−5.86–−0.90	+
H:prf/aso	18	0.64	1.29	0.87–1.92	−1.12	−2.24–−0.37	iso
H:aso/pso	16	0.68	1.85	1.23–2.78	−2.21	−3.85–−1.11	+
H:pso/po	17	0.72	1.45	0.99–2.10	−1.32	−2.48–−0.52	iso
T:fp	24	0.7	1.23	0.90–1.67	−0.58	−1.38–−0.00	iso

Abbreviations as in Table 2.

**Table 13:** Allometric regression of *Stegoceras validum* frontoparietal heights against frontoparietal length excluding all specimens less domed (thick) than TMP 84.5.1.

Frontoparietal Length							
	N	R	Slope	CI	Intercept	CI	Allometry
H:n/n	8	0.77	1.43	0.78–2.61	−1.6	−3.91–−0.34	iso
H:n/prf	6	0.75	1.32	0.58–2.98	−1.47	−4.74–−0.03	iso
H:prf/aso	9	0.89	1.48	1.00–2.19	−1.78	−3.18–−0.84	iso
H:aso/pso	7	0.94	2.05	1.42–2.97	−3.01	−4.79–−1.78	+
H:pso/po	9	0.71	1.4	0.77–2.54	−1.56	−3.79–−0.32	iso
T:fp	9	0.69	1.66	0.90–3.05	−1.69	−4.43–−0.20	iso

Abbreviations as in Table 2.

**Table 14:** Allometric regression of *Stegoceras validum* frontoparietal heights against parietal length excluding all specimens less domed (thick) than TMP 84.5.1.

Parietal Length							
	N	R	Slope	CI	Intercept	CI	Allometry
H:n/n	8	0.86	1.15	0.70–1.87	−0.73	−1.94–0.01	iso
H:n/prf	6	0.77	1.11	0.50–2.47	−0.75	−3.03–0.28	iso
H:prf/aso	9	0.81	1.16	0.70–1.92	−0.84	−2.13–−0.06	iso
H:aso/pso	9	0.94	1.44	0.99–2.11	−1.43	−2.54–−0.67	iso
H:pso/po	9	0.84	1.1	0.69–1.74	−0.66	−1.76–0.03	iso
T:fp	11	0.82	1.35	0.88–2.05	−0.73	−1.93–0.06	iso

Abbreviations as in Table 2.

**Table 15:** Allometric regression of *Stegoceras validum* frontoparietal heights against frontal length excluding all specimens less domed (thick) than TMP 84.5.1.

Frontal Length							
	N	R	Slope	CI	Intercept	CI	Allometry
H:n/n	14	0.15	2.23	1.24–4.01	−2.46	−5.38–−0.83	+
H:n/prf	12	−0.08	−2.7	−5.19–−1.40	5.56	3.44–9.66	
H:prf/aso	16	0.24	2.29	1.35–3.89	−2.61	−5.24–−1.06	+
H:aso/pso	14	0.05	3.79	2.09–6.86	−5.15	−10.16–−2.38	+
H:pso/po	16	0.08	2.32	1.35–4.00	−2.59	−5.34–−0.99	+
T:fp	18	0.34	2.48	1.54–4.01	−2.49	−5.00–−0.93	+

Abbreviations as in Table 2.

**Table 16:** Allometric regression of *Stegoceras validum* frontoparietal heights against frontoparietal width excluding all specimens less domed (thick) than TMP 84.5.1, except for three partially domed frontals (TMP 78.19.04, 86.71.2, and 2002.12.57).

Frontoparietal Width							
	N	R	Slope	CI	Intercept	CI	Allometry
H:n/n	15	0.74	1.9	1.28–2.82	−2.13	−3.73–−1.05	+
H:n/prf	14	0.63	2.08	1.30–3.31	−2.48	−4.62–−1.14	+
H:prf/aso	21	0.69	1.43	1.02–2.01	−1.38	−2.39–−0.65	+
H:aso/pso	19	0.74	1.76	1.25–2.47	−2.04	−3.27–−1.15	+
H:pso/po	20	0.8	1.55	1.16–2.08	−1.51	−2.44–−0.82	+
T:fp	27	0.72	1.64	1.24–2.17	−1.33	−2.27–−0.63	+

Abbreviations as in Table 2.

**Table 17:** Allometric regression of *Stegoceras validum* frontoparietal heights against frontal length excluding all specimens less domed (thick) than TMP 84.5.1, except for three partially domed frontals (TMP 78.19.04, 86.71.2, and 2002.12.57).

Frontal Length							
	N	R	Slope	CI	Intercept	CI	Allometry
H:n/n	15	0.33	3.3	1.92–5.65	−4.23	−8.09–−1.98	+
H:n/prf	13	0.14	3.39	1.83–6.29	−4.45	−9.20–−1.89	+
H:prf/aso	17	0.36	2.72	1.66–4.46	−3.33	−6.18–−1.59	+
H:aso/pso	15	0.19	4.11	2.35–7.19	−5.68	−10.70–−2.81	+
H:pso/po	17	0.24	2.79	1.67–4.65	−3.36	−6.41–−1.53	+
T:fp	19	0.44	3.08	1.98–4.81	−3.49	−6.32–−1.68	+

Abbreviations as in Table 2.

The height of the dome generally exhibits positive allometric growth (Tables 2, 3, 4, 5, Fig. 5A). The allometry is strongest when compared to frontoparietal width and frontal length, where the slopes are all significantly positive. In all cases, the thickness of the frontoparietal shows strong positive allometry (contra Chapman et al. [21]). When compared to frontoparietal length and parietal length the slopes are generally lower, although in most cases the difference is not significant. The slopes for the heights are considerably higher when frontal length is used as the standard variable. This is likely due to the negative allometry of frontal length when compared to the other standard variables (see below), but also may be affected by the lower correlation coefficients.

The growth of the width of the dome does not show a single definitive pattern (Tables 6, 7, 8, Figure 5B). When compared to frontoparietal width, some of the dome widths exhibit isometry (W:n/prf and W:po/stf/sq) and others show significant negative allometry (W:prf/aso, W:aso/pso, W:pso/po). All of the widths show isometry when frontoparietal length is used as the standard variable. The same is true when parietal length is used as the standard, except that W:fp is positively allometric.

The lengths of the frontoparietal sutures all exhibit statistically isometric growth and in most cases the slopes are near one (Tables 9, 10, 11, Fig. 5C). Both the frontal and parietal lengths show negative allometry when compared to frontoparietal width (Table 9, Fig. 5D). The length of the frontoparietal is statistically isometric, but the slope is rather low (0.67), and thus detection is likely hindered by the small sample size (n = 10). Additionally, frontal length is negatively isometric when compared to both frontoparietal and parietal lengths.

When flat-headed specimens, the holotype of *Ornatotholus browni*, and partially domed frontals are excluded from the bivariate analysis, allometric regression of frontoparietal heights generally resulted in lower slopes (Tables 12, 13, 14, 15, Fig. 6). The general trends, however, were similar in all comparisons to those using the total sample, although with the smaller sample most of the results did not differ significantly from isometry. The exception to this is comparisons based on frontal length, which showed positive allometry, but the low correlations suggest the results are not meaningful. For frontoparietal thickness compared to frontoparietal width or length, the slopes remained positive, but were lower and not statistically different from isometry. It appears that the small specimens anchor the regression line, and may also suggest that the slope decreases for larger frontoparietals; however the small sample size of large sized domes prevents us from further addressing this possibility.

When the three partially domed frontals (TMP 78.19.4, TMP 86.71.2, and TMP 2002.12.57) were added back into the dataset, only statistically positive allometric relationships were recovered when frontoparietal heights are regressed against width (Tables 16 and 17, Fig. 6). In most cases, the slopes are not significantly different than when all specimens are included in the analysis, with the exception of frontoparietal thickness, which was significantly lower.

### Frontoparietal Bone Histology

#### Morphological Comparisons

**Figure 7:**
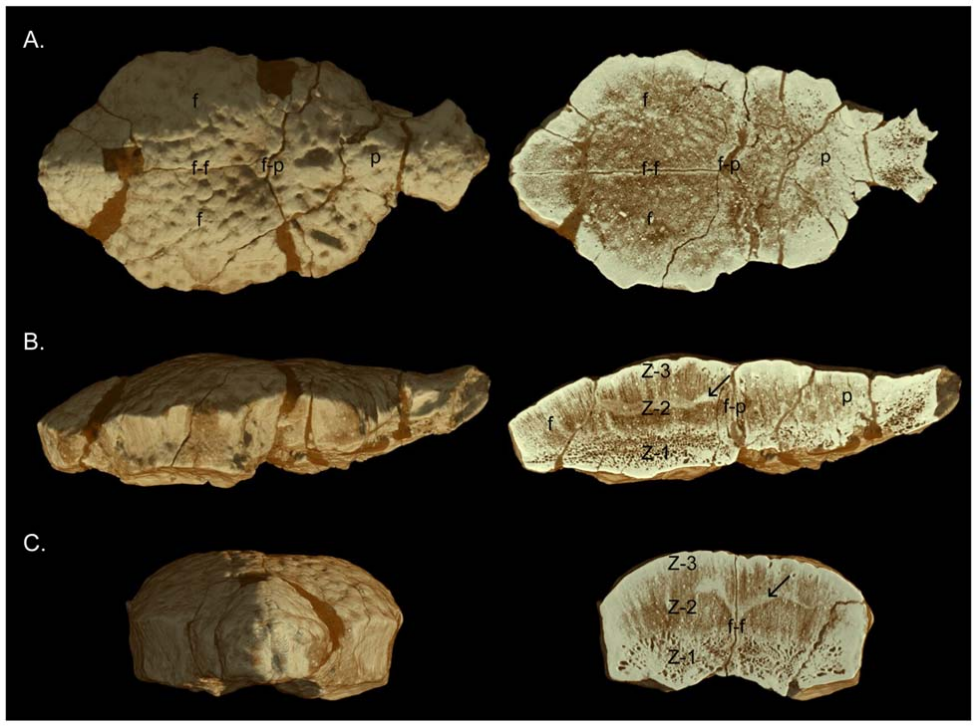
High-resolution CT images of AMNH 5450. **A**, dorsal view (left) and frontal section (right); **B**, lateral view (left) and sagittal section (right); **C**, anterior view (left) and transverse section through the frontal (right). The histological zones of Goodwin and Horner [37] are denoted. The arrow identifies an artifact likely produced by mineralized collagen fibres present across and along the interfrontal suture. Abbreviations: **f**, frontal; **f-f**, inter-frontal suture; **f-p**, frontoparietal suture; **p**, parietal; **po**, postorbital; **pso**, posterior supraorbital; **sq**, squamosal; **Z-1** to **Z-3**, histological Zones I to III.

Reconstructions and slices from the HRCT scans of AMNH 5450, the smallest scanned specimen, are shown in Figure 7. Plaster-filled breaks along the frontoparietal suture and anterior frontal, nearly indistinguishable on the original, are clearly visible by the darker brown colour. In dorsal view (Fig. 7A), the low-relief tubercular ornamentation and the interfrontal and frontoparietal sutures, which are filled with plaster, are evident. In sagittal section (Fig. 7B), the contrasting vascularity within Zones I–III is visible. The plaster-filled frontoparietal suture is traceable from the roof of the braincase to the dorsal surface of the frontoparietal. The slightly ‘wavy’ brighter layer in the upper region of Zone II is likely an artifact produced by mineralized collagen fibres present across and bordering the interfrontal suture. A horizontal slice from above the roof of the braincase through the highly vascular Zone II reveals the interfrontal and plaster-filled frontoparietal sutures (Fig. 7A). Anteriorly, the prefrontal-frontal suture is partially obscured by plaster. In anterior view, the interfrontal suture is visible along the midline of the frontal and is tightly interdigitated anteriorly (Fig. 7C). The transverse slice through the frontals (Fig. 7C) shows the open interfrontal suture, which extends from the roof of the braincase to the dorsal surface of the thickened but undomed frontal. The internal microstructure is zonal and a slightly truncated ‘m’-shaped layer in Zone II appears as a brighter coloured region with relatively less vascularity. This appears to be the same HRCT artifact produced by the presence of mineralized connective tissue or bundles of collagen fibres present along patent cranial sutures.

**Figure 8:**
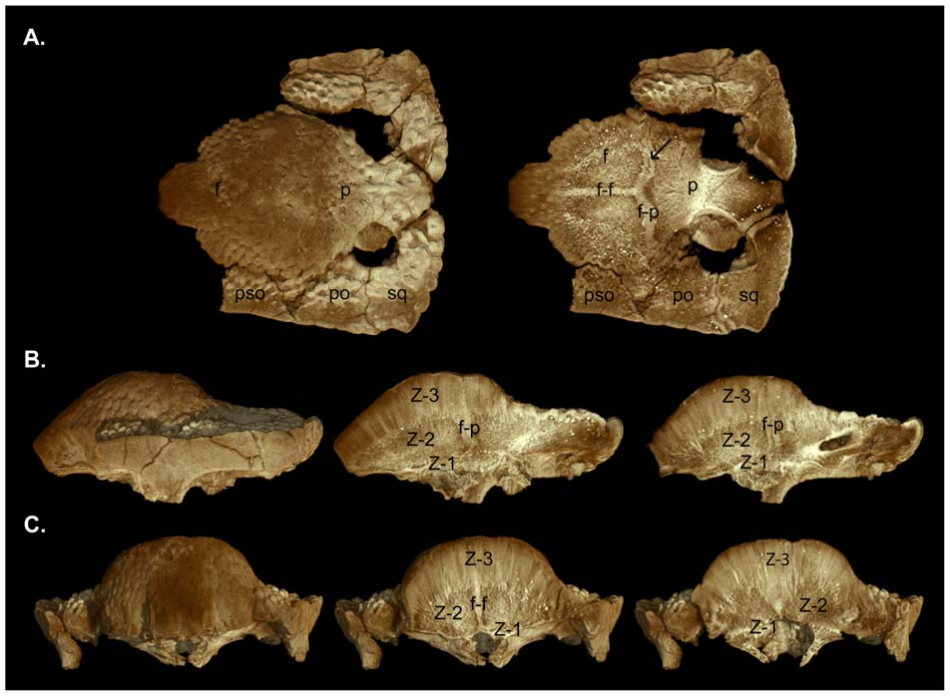
High-resolution CT images of TMP 84.5.1. **A**, dorsal view (left) and frontal section (right); **B**, lateral view (left), median sagittal section (middle), and lateral sagittal section (right); **C**, anterior view (left), anterior transverse section through the frontal (middle), and posterior transverse section through the parietal (right). The histological zones of Goodwin and Horner [37] are denoted. The arrow identifies an artifact likely produced by mineralized collagen fibres present across and along the interfrontal and frontoparietal sutures. Abbreviations: **f**, frontal; **f-f**, inter-frontal suture; **f-p**, frontoparietal suture; **p**, parietal; **po**, postorbital; **pso**, posterior supraorbital; **sq**, squamosal; **Z-1** to **Z-3**, histological Zones I to III.

Reconstructions and corresponding slices of TMP 84.5.1 are shown in Figure 8. The reconstruction of the left lateral view (Fig. 8B) confirms that the inflated frontal and anterior parietal contribute to the cranial dome at this ontogenetic stage. The dorsal surface is covered by tubercular ornamentation and the posterior parietal is thickened but undomed. Sutures between the lateral cranial elements are visible. A trace of the frontoparietal suture occurs as a ‘blurry’ region on the dorsal surface, but is effectively indistinguishable in the fossil. In sagittal section (Fig. 8B), the highly vascular tissue of the interior skull is apparent, along with the braincase and patent cranial sutures. Zone II appears to either abruptly end in the middle of the skull (Fig. 8B middle) or exhibit a visible texture change where it contacts a layer of what appears to be relatively denser, radiating tissue (Fig. 8B right). We interpret this change in texture as an artifact of HRCT produced by the increase in concentration or change in orientation of mineralized collagen fibres along the interfrontal and frontoparietal sutures. Higher resolution histology thin-section slides unmistakably show the continuation of Zone II nearly to the edge of the frontal dome in pachycephalosaurs of this ontogenetic stage (see Goodwin and Horner [37] Fig. 4B, Fig. 5C).

The frontoparietal suture is visible as a sinuous darker vertical line bordered by lighter-coloured tissue denoting this intermembranous bone growth site in the skull (Fig. 8B right). The interfrontal and frontoparietal sutures are not visible on the dorsal surface of the skull because they are tightly closed, obscuring the relatively early developmental stage of the frontoparietal dome (Fig. 8A). Sutures between the supraorbital, postorbital, and squamosal are patent and, along with the modest frontoparietal dome and open supratemporal fenestrae, support the juvenile ontogenetic age assignment of this skull.

A horizontal slice above the roof of the braincase reveals the increased vascularity of Zone II within the frontoparietal dome and patent cranial sutures throughout the skull (Fig. 8A). The lighter coloured zones bordering the patent interfrontal, the interdigitated frontoparietal, and associated cranial sutures are likely an HRCT artifact produced by the abundant mineralized collagen (Sharpey's) fibres present along these contacts. The anterior transverse section of TMP 84.5.1 (Fig. 8B) shows the interfrontal suture dividing Zone II nearly in half along the midline where it contacts the dorsal roof of the braincase. Differences in the relative vascularity between Zones I, II, and III are visible, but the boundaries between these zones are not as clearly defined using HRCT as they are in histological slides at this stage of ontogeny (see Goodwin and Horner [37] Fig. 5C). Zone II extends nearly to the dorsal surface of the highly expanding, fast growing frontoparietal dome in this stage of ontogeny. This extension of Zone II is obscured by the HRCT artifact giving this bone a denser appearance or texture compared with the surrounding more vascular tissue. The more posterior coronal section shows the HRCT produced artifact of asymmetry in Zone II that is caused by the presence of mineralized collagen (Sharpey's) fibres concentrated along the frontoparietal and interfrontal sutures.

**Figure 9:**
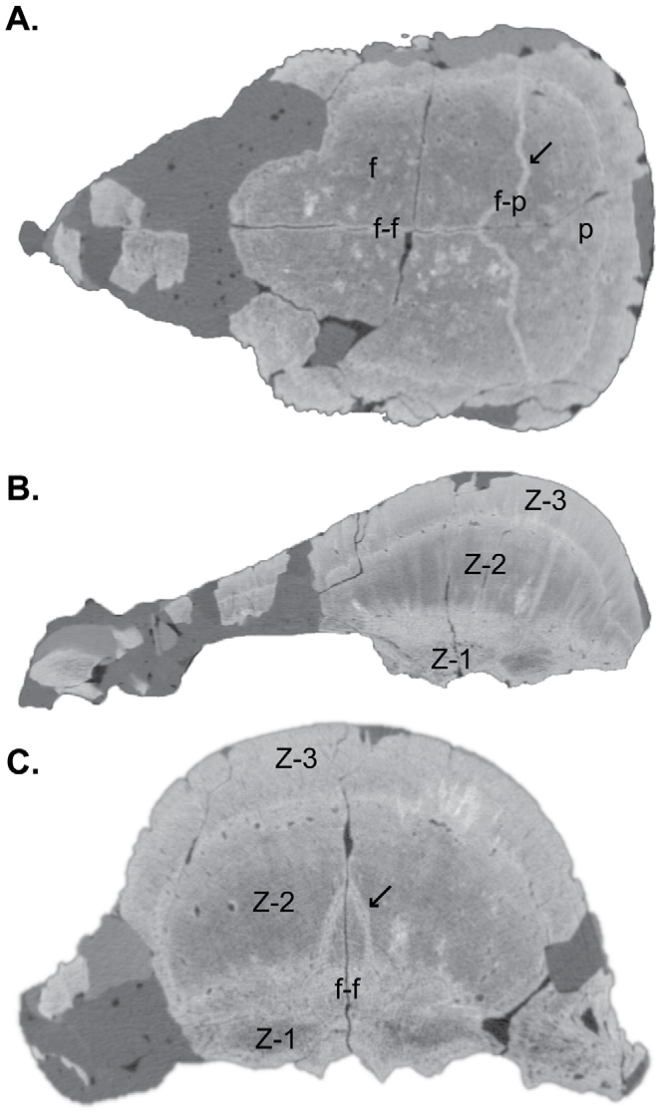
High-resolution CT images of ROM 53555. **A**, frontal section; **B**, median sagittal section; **C**, transverse section through the posterior portion of the frontal. The histological zones of Goodwin and Horner [37] are denoted. The arrow identifies an artifact likely produced by mineralized collagen fibres. Plaster reconstructions are visible as dark grey areas. Abbreviations: **f**, frontal; **f-f**, inter-frontal suture; **f-p**, frontoparietal suture; **p**, parietal; **Z-1** to **Z-3**, histological Zones I to III.

Frontal, sagittal, and transverse sections from CT scans of ROM 53555 are shown in Figure 9. The frontal section (Fig. 9A) reveals that both the interfrontal and frontoparietal sutures remain open internally even among the largest, and presumably oldest, known individuals of *Stegoceras validum*. While the frontoparietal suture remains bordered by the lighter coloured zone at this stage, the inter-frontal suture no longer is. In both the sagittal and the transverse sections (Fig. 9B,C) the three histological Zones are clearly distinguishable, but the lighter-coloured ‘wavy’ band that was found through Zone II appears to have expanded and now separates Zones I and II. This lighter-coloured band continues to extend upwards into Zone II in the transverse section (Fig. 9C, arrow). Additionally, in the transverse section the open interfrontal suture is visible. Overall, these sections show less vascular canals than the smaller specimens. However, Zone I and II are still distinguishable as being less dense than Zone III based on the darker colour in the scan. The CT scans of UALVP 2 (not shown) compare well to ROM 53555, suggesting a similar histological development in the two specimens.

#### Relative Vascularity

**Figure 10:**
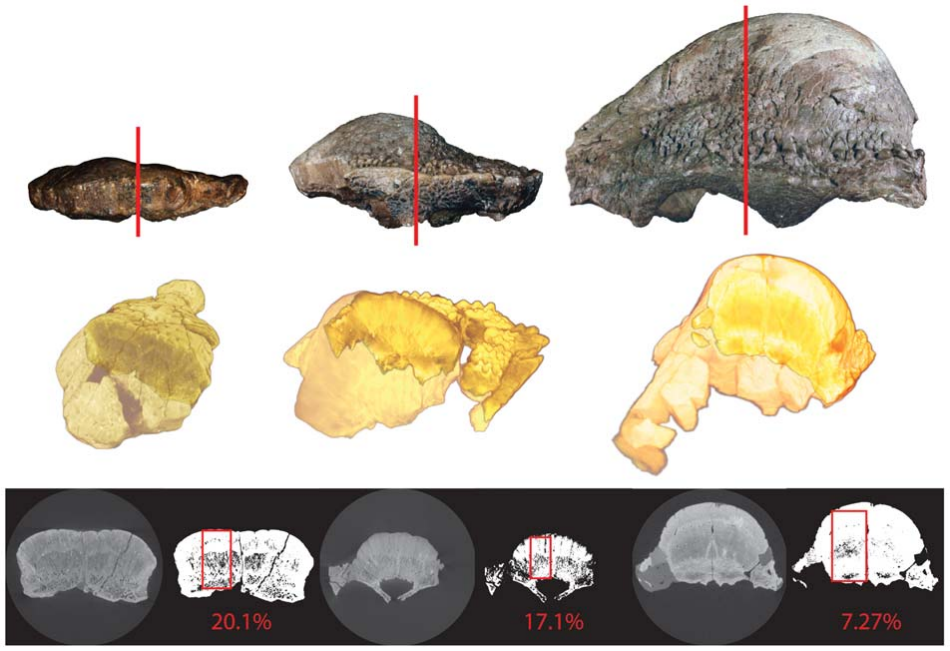
Outline of methodology for calculation of relative void-space. AMNH 5450 (left), TMP 84.5.1 (middle), and ROM 53555 (right). A transverse section was taken from a HRCT scan of the frontal at the contact of the posterior supraorbital and postorbital sutures. Scans were thresholded using the Huang method [54], [55]. Void-space was calculated in a region of interest (red square).

**Figure 11:**
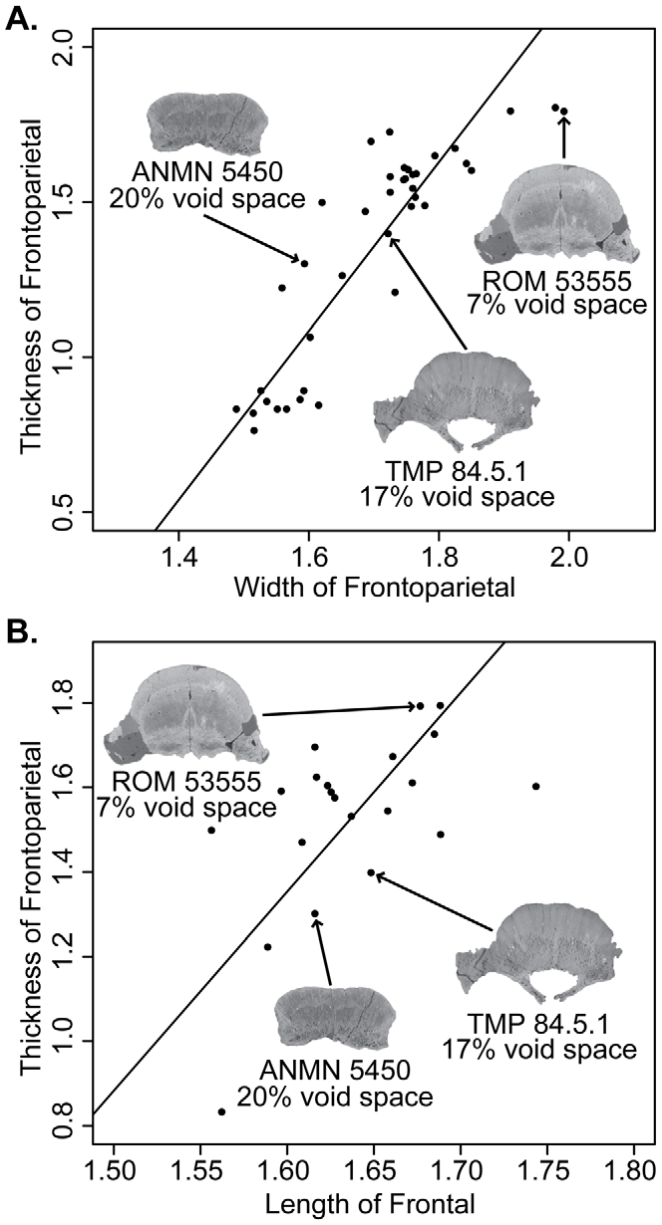
Bivariate logarithmic plots with RMA regression lines for frontoparietal thickness vs width (A) and frontoparietal thickness vs frontal length (B) and the CT scans for AMNH 5450, TMP 84.5.1, and ROM 5355 and their relative void-spaces (a proxy for vascularity). Note the substantial decrease of vascularity with increased dome development (thickness) and size (frontoparietal width, A; frontal length, B). Measurements are log (mm).

The specimens and slices used to calculate relative vascularity are illustrated in Figure 10. Relative void space (our proxy for relative vascularity) decreased with each successively larger specimen from 20% in AMNH 5450, to 17% in TMP 84.5.1, to 7% in ROM 53555. This is further illustrated in Figure 11, where relative void space is plotted with regressions of frontoparietal thickness on frontoparietal width and frontal length, respectively. Additionally, our rough estimate of relative void-space in UALVP 2, which of a similar size to ROM 53555, was ∼7%.These data clearly show that a decrease in relative vascularity correlates with the development of the frontoparietal dome, as predicted by the ontogenetic model of Goodwin and Horner [37].

## Discussion

### Frontoparietal Ontogeny of *Stegoceras validum*

The recognition of undomed individuals exhibiting diagnostic squamosal ornamentation of *Stegoceras validum*, together with allometric and histological proxy data, provides strong corroboration of the hypothesis that the skull of *S. validum* underwent marked changes in shape during growth, with flat-headed juveniles developing into adults with highly domed skulls. Contrary to the results of Chapman et al. [21], our bivariate analyses show that frontoparietal thickness exhibits significant positive allometry with respect to frontoparietal width and length, a result fully consistent with a growth series from a flat-headed to a fully domed morphology. While the regression slopes decreased when flat-headed specimens were excluded from the analysis, this result is likely explained by the substantial decrease in sample size and range. A decrease in growth rates with increased size, as found with the positive allometry of beetle horns [60], may also be a complicating factor in this respect. Unfortunately, the current sample size of large specimens is too small to test this further.

The morphometric analysis reveals several notable changes in the shape of the frontoparietal through the ontogeny of *Stegoceras validum*. The width of the frontoparietal is positively allometric with respect to its length, and the width across the frontoparietal suture increased at a relatively faster rate than the width across the supraorbital lobes. Additionally, the length of the parietal increased at a faster rate than that of the frontal. These changes modify the shape of the frontoparietal throughout ontogeny beyond that expected from simple thickening. As the frontoparietal increases in size, the dome becomes relatively thicker and wider posteriorly, resulting in the distinctive pear-shaped dome that characterizes *Stegoceras*. A similar growth pattern may also characterize *Colepiocephale*, given the similar shape of its cranial dome [5]. These growth-related changes in the proportions of the frontoparietal suggest that the use of frontoparietal ratios in systematic assessments of specimens and in phylogenetic analyses [17] should be considered carefully.

HRCT scans of the growth series show a marked decrease in relative void space (a proxy for vascularity) coinciding with the development of the dome, a pattern which was first proposed in pachycephalosaurs using traditional, destructive thin-section techniques. The new techniques developed here for documenting and quantifying changes in relative vascularity make it possible to assign relative ontogenetic ages to specimens without the need to destroy them for histological assessment. This methodology can be extended to other pachycephalosaur taxa and used to test conflicting hypotheses of synonymy and alpha taxonomy [1], [9], [10]. Additionally, comparisons of histology with other pachycephalosaur taxa will likely shed additional light on ontogenetic patterns and hypotheses of dome function in this group.

Numerous morphological changes accompany doming throughout the cranial ontogeny of *Stegoceras* (Table 18), some of which were hypothesized previously by Williamson and Carr [10]. One notable difference between adults and juveniles is the fusion of the frontals to each other, and to the parietals. Fusion appears to occur first between the frontals, and then between the co-ossified frontals and the parietals. For example, in CMN 3135 and CMN 38428 the inter-frontal suture is not visible dorsally or ventrally, but a well-developed frontoparietal suture is retained. However, the HRCT scans show that fusion occurs in the outer (external) layers of bone while the frontoparietal and interfrontal sutures remain open internally, even in the largest specimens (ROM 53555, Fig. 9), indicating the potential for continued cranial growth.

**Table 18:** Ontogenetic changes in the cranial morphology of *Stegoceras validum*.

Juvenile	Adult
• Frontals and parietal flat	• Frontals and parietal thickened and domed
• Dorsal surface of frontals and parietal covered in numerous tubercles	• Frontoparietal smooth, tubercles present only on periphery
• Frontal-frontal suture open	• Frontals fused externally
• Frontoparietal suture open	• Frontaloparietals fused externally
• Frontoparietal highly vascular	• Vascularity highly reduced
• Frontoparietal rectangular	• Frontoparietal pear-shaped
• Posterior and lateral shelves flat and prominent	• Posterior and lateral shelves reduced and incorporated into the dome
• Posteromedial extension of parietal long and flat	• Posteromedial extension of parietal short and mostly incorporated into dome
• Postorbitals and posterior supraorbitals flat	• Postorbitals and posterior supraorbitals inflated and contribute to dome
• Frontonasal boss flat	• Frontonasal boss high, convex and separated by grooves

The peripheral elements of the skull table that contact the frontoparietal also change shape substantially through ontogeny: the posterior (parietosquamosal) and lateral shelves formed by the parietal, squamosals, postorbital, and supraorbitals are shortened, relative to overall size, as they become incorporated into the dome during ontogeny. This results from the inflation of the medial extension of the parietal, the postorbitals, and the posterior supraorbitals as they become incorporated into the dome. The anterior supraorbital bones and the supraorbital lobe of the frontal do not overly inflate to form a significant contribution to the dome in adult *Stegoceras validum* (Fig 5A). This results in deep grooves separating the supraorbital lobes from a prominent frontonasal boss, a feature that is characteristic of *Stegoceras* and other closely related taxa.

On the basis of their morphometric analysis, Chapman et al. [21] inferred sexual dimorphism in *Stegoceras validum*. Although we did not test for this explicitly, our new analysis, which includes only *Stegoceras* specimens (sensu Sullivan [9]) from the upper Belly River Group of Alberta, did not reveal any compelling evidence of sexual dimorphism in dome shape based on visual inspection of the plots. The available sample of large individuals is small, making the identification of bimodal trends or clusters difficult. Allometric patterns based on our sample suggest a significant amount of variation in the growth trajectory of the dome, but this appears to be largely size-related with no clear evidence of any dimorphic trends. Therefore, it is likely that the inferences of Chapman et al. [21] with respect to sexual dimorphism were influenced by time averaging and the inclusion of specimens from other distinct taxa (e.g., those of ‘*Prenocephale*’ *brevis* and *Colepiocephale lambei*), in addition to any affects associated with the lack of morphological landmarks, a methodological flaw identified by Goodwin [23]. While we found no evidence of sexual dimorphism in the shape of the frontoparietal, this does not preclude the possibility of sexual size dimorphism with conserved relative growth relationships. Further histological sampling is required to assess this possibility.

### HRCT and Presence of Collagen Fibres along Cranial Sutures

HRCT reveals morphological and sutural details that are not easily distinguishable on the surface of pachycephalosaur skulls due to relative ontogenetic stage, preservation, or excess plaster and consolidants during preparation. HRCT confirms the interfrontal and frontoparietal sutures, reconstructed plaster regions, and glued breaks in the holotype of *Ornatotholus browni* ( = *Stegoceras validum*; AMNH 5450). In the partial skull of a juvenile *S. validum*, TMP 84.5.1, vascular zones and cranial sutures are clearly seen, however the boundaries between these zones are less clear than revealed by standard histological slides on account of an HRCT-produced artifact from the presence of mineralized collagen (Sharpey's) fibres along cranial sutures. The arrangement and orientation of these collagen fibres and the corresponding ectocranial suture sinuosity and cross-sectional interdigitization reflect loading and skull deformation properties in mammals [61], [62] and fish [63].

These fibrous joints or sutural contacts between the bones of the skull are linked together by collagen fibres and connective tissue that bridge the contacting cranial bones [62]. In mammals, these sutures are the site of intramembranous bone growth in the skull and the major centre of bone expansion within the craniofacial vault [64]. New bone is produced at the sutural edges of the bone front in response to external stimuli during cranial morphogenesis. There is evidence that the frontoparietal domes in pachycephalosaurs, and cranial ornamentation in most dinosaurs, grew much differently than the cranial bones in mammals and other tetrapods [16], [65]. In the HRCT scans of pachycephalosaurs presented here, these intermembranous bone growth sites appear to be relatively denser (lighter colour) compared to the surrounding tissue in the artificially coloured slices from the skull (Figs. 7 and 8). We hypothesize that this HRCT produced artifact is due to the preservation of dense concentrations of mineralized collagen (Sharpey's) fibres along these cranial sutures visible in histological slides of *Stegoceras* [37]. Alternatively, the features and structure of these sutural contacts and surrounding tissue may also be related to aspects of metaplastic fibrous deposition in the Marginocephalia [16]. The boundaries between Zones I–III defined by Goodwin and Horner [37] are also less distinct in the HRCT slices, however Zone I and II are still highly vascular compared to Zone III.

### Implications for Flat-headed Pachycephalosaur Taxa

The presence of a transitional flat-headed morphology in *Stegoceras validum* has significant implications for the delineation of pachycephalosaur species. Recent work by a number of authors has called into question the validity of all flat-headed taxa, with the potential that they may be immature specimens, many of which may pertain to previously named taxa [16], or be paedomorphic in nature [20]. Due to large samples from a relatively restricted stratigraphic interval, the reconstructed ontogenetic series of *S. validum* in this study serves as the most complete model for testing and confirming ontogenetic variation in pachycephalosaurs.

Although many workers have previously considered *Ornatotholus browni* to represent a juvenile of *Stegoceras validum* [9], [10], [15], [40], Sereno [8] and Maryańska et al. [1] recognized *O. browni* as a distinct taxon in their reviews of Pachycephalosauria without justification or comments on its diagnostic characters or ontogenetic status. The holotype of *O. browni* was diagnosed on three characters of the frontoparietal, each of which is discussed below.

The tuberculate surface texture identified as diagnostic of *Ornatotholus browni* by Galton and Sues [14] covers the dorsal surface of the frontal, parietal, supraorbitals, postorbital, and squamosal of UCMZ(VP) 2008.001, but a similar texture also covers the uninflated portions of the dome of *Stegoceras* [10] and other pachycephalosaurs (e.g., *Homalocephale*, *Goyocephale, Wannanosaurus*, *Dracorex*, *Stygimoloch*). Furthermore, the squamosal and postorbital of domed *S. validum* specimens (e.g., TMP 84.5.1) have a surface texture that is identical to that in flat-headed specimens (UCMZ(VP) 2008.001 and UALVP 49531). Thus, tuberculate surface texture appears to be a, presumably plesiomorphic, feature of known pachycephalosaurs that is associated with uninflated portions of the skull [10], [37].

In a comparison of AMNH 5450 with the small *Stegoceras* specimen CMN 138, Galton and Sues [14] diagnosed *Ornatotholus browni* on the basis of having supratemporal fenestrae that were larger than the fenestrae seen in *S. validum*. While the conclusion of Williamson and Carr [10] that the supratemporal fenestrae close with ontogeny may be generally correct, there is a high amount of variation in supratemporal fenestrae size that shows little correlation with frontoparietal size. UCMZ(VP) 2008.001 has a maximum supratemporal diameter of 11.6 mm, whereas TMP 84.5.1, a domed specimen of *S. validum* has an average maximum supratemporal fenestra diameter of 16.5 mm. This is much larger than would be expected if the supratemporal fenestrae simply closed with ontogeny. CMN 138, another domed specimen of *S. validum*, and about the same size as TMP 84.5.1, has an average maximum supratemporal fenestra diameter of 9.1 mm. In addition to variation in relative size between specimens, the supratemporal fenestrae also show marked asymmetry within specimens, notable in UALVP 2, and exemplified by CMN 8816 where the left fenestra appears to be closed, but the right is open [12]. Clearly, variation in the size and degree of closure of the supratemporal fenestrae is more complex than presently understood, and may simply exhibit a high degree of individual variation independent of ontogeny. Further study will be needed to clarify the significance of variation in this character.

The final character used to diagnose *Ornatotholus browni* is the presence of a low dome divided by a shallow transverse depression [14]. A shallow transverse depression is not found in other specimens referred to the genus (e.g., TMP 78.19.4, UCMP 130295), or in the other known flat frontals or parietals (e.g., UCMZ(VP) 2008.001, UCMZ 2008.002, TMP 78.19.16), nor is it found in any other pachycephalosaur specimen known. Rather than a diagnostic character, we concur with Williamson and Carr [10] and Goodwin et al. [15] that this depression most likely represents a transitory ontogenetic feature related to the initiation of dome growth. Early in dome ontogeny, doming may begin separately in the frontals and parietal, producing a shallow transverse depression. This depression likely reflects the growth plate in the dermal bone that is obliterated from external view as the frontal and parietal domes combine to form a single dome. Evidence for this feature is also found in another partially inflated frontal, TMP 2002.12.57, that is approximately the same size as AMNH 5450. In TMP 2002.12.57, the dome curves ventrally just anterior to the sutural surface for the parietal and thus would have formed a similar transverse depression along the frontoparietal suture. This character is not present in either flat or more fully domed specimens and is considered here to be a transitory ontogenetic feature rather than a diagnostic character.

The flat-headed pachycephalosaur taxon *Wannanosaurus* has been considered a juvenile based on unfused cranial sutures, large supratemporal fenestrae, and nodular surface texture [8], [65], all characters that are shared with juvenile *Stegoceras* and are reduced and eventually eliminated during ontogeny in the latter taxon. *Goyocephale*, *Homalocephale*, and *Dracorex*, the remaining flat-headed taxa, also share these three characteristics [19], [58], [66], [67], and thus may also represent juveniles. However, open cranial sutures and supratemporal fenestrae are plesiomophic within Ornithischia, and should be expected in the adult stage of a primitive, undomed member of the pachycephalosaur lineage. Resolution of the ontogenetic and taxomonic status of flat-headed pachycephalosaurs will require the use of multiple independent lines of evidence, as developed in this study. Although it is possible that histological examination could reveal the hallmarks of maturity in currently known flat-headed specimens, these issues will likely only be resolved definitively with larger sample sizes, which requires the discovery of new material [19].

### Conclusions

This study shows that as *Stegoceras* matured, the skull changed shape dramatically, and demonstrates conclusively that *Ornatotholus browni* represents a transient ontogenetic stage of *S. validum*. The extensive nature of these changes is such that juveniles and adults differ radically in their general appearance, and we hypothesize that this model of dome growth is a common developmental trajectory of domed pachycephalosaurs. The phenomenon of extreme morphological differences between juveniles and adults is becoming increasingly well-documented in ornithischian dinosaurs, including hadrosaurs [68], [69] and ceratopsids [70]–[72]. Historically, these transitional juvenile morphologies have been erected as distinct taxa, resulting in artificially inflated estimates of biodiversity in these groups (e.g., [72], [73]) and complicating phylogenetic studies. Hypotheses of extreme ontogenetic change in a number of dinosaurs, such as *Triceratops* [74]–[76] and *Pachycephalosaurus* [16], [66], remain controversial. It is important to understand the relationship of morphology to size and other ontogenetic influences in assessing the nature of variation and taxonomic composition in a given sample. This study emphasizes the need for large sample sizes and an integrative approach utilizing multiple independent lines of evidence for testing competing hypotheses of extreme ontogenetic change or taxic delineation in pachycephalosaurs and other dinosaurs.

## Supporting Information

Table S1 **Specimens of** ***Stegoceras validum*** **used in the allometric analyses and their measurements.**
(DOC)

Text S1 **Description of measurements.**
(DOC)

Text S2 **HRCT methods.**
(DOC)
