# Supplementary material for: Cranial Ontogeny in Stegoceras validum (Dinosauria: Pachycephalosauria): A Quantitative Model of Pachycephalosaur Dome Growth and Variation
Source: PLoS One. 2011 Jun 29;6(6):e21092. doi: 10.1371/journal.pone.0021092 (PMC3126802; doi:10.1371/journal.pone.0021092)
Supplement: Table S1 — Specimens of Stegoceras validum used in the allometric analyses and their measurements. (DOC) [file pone.0021092.s001.doc]

**Table S1. Specimens of *Stegoceras validum* used in the allometric analyses and their measurements.**

| Specimen | H:n/n | H:n/prf | H:pfr/aso | H:aso/pso | H:pso/po | W:n/pfr | W:pfr/aso | W:aso/pso |
| --- | --- | --- | --- | --- | --- | --- | --- | --- |
| AMNH5450 | 9.9 | 9.4 | 11.7 | 11.4 | 14.3 | 18.4 | 30.7 | 43.8 |
| BMNH-R.-8673 |  |  |  |  |  | 21.1 | 44.9 |  |
| BMNH-R.-8674 |  |  |  |  |  |  |  |  |
| CMN1108A | 17.4 | 14.9 | 16.3 | 13.5 | 17.1 | *20.0* | 37.1 | *50.0* |
| CMN138 | 15.2 | 10.1 | 11.2 | 8.9 | 14.3 | 25.8 | 39.4 | 55.1 |
| CMN1594 |  |  |  |  |  |  |  |  |
| CMN2379 | *32.2* | *19.0* | *15.5* | *14.5* | *22.0* |  | 42.4 |  |
| CMN38428 | 19.4 | 16.9 | 17.2 | 7.6 | *21.0* | 27.3 | 53.4 | 66.6 |
| CMN515 | 16.5 | 16.0 | 15.4 | 10.2 | 14.7 | 26.5 | 41.9 | 51.8 |
| CMN8816 | 11.6 | 10.2 | 11.4 | 8.5 | 14.2 |  | 36.4 | 50.0 |
| ROM58311 | 17.3 |  | 11.4 | 9.9 | 18.5 | 29.4 | 47.8 | 60.9 |
| ROM803 |  |  | 14.7 | 8.5 |  | 25.3 | 39.1 | 51.7 |
| ROM53555 |  |  |  |  |  |  |  |  |
| TMP2000.26.1 |  |  | *23.8* | *24.1* | 34.4 |  |  |  |
| TMP2002.12.57 | 5.8 | 5.5 | 7.1 | 5.7 | *8.2* | *17.8* | *29.0* | *40.4* |
| TMP75.11.5 |  |  |  |  |  | 23.9 | 37.2 | 50.6 |
| TMP78.19.04 |  |  | 7.8 | 7.8 | 11.4 |  | *50.3* | *65.4* |
| TMP81.41.102 | 2.8 | 4.3 | 3.1 | 4.1 | 6.5 | *20.0* | *35.0* | *41.0* |
| TMP82.20.189 |  |  |  |  |  |  |  |  |
| TMP83.67.1 | *21.2* | *14.2* | *13.1* | *7.2* | *13.6* | 29.4 | 45.3 | 59.7 |
| TMP84.05.01 | 13.6 |  | 11.7 | 7.8 | 12.6 | 21.2 | 36.3 | 48.6 |
| TMP85.58.68 |  |  |  |  |  |  |  | 37.4 |
| TMP86.71.2 |  | *11.2* | 12.3 | 9.5 | *10.3* |  | *30.4* | *44.4* |
| TMP88.116.49 |  |  |  |  |  |  |  |  |
| TMP92.02.36 | 12.3 | 12.0 | 10.7 | 7.1 | 12.9 | 18.2 | 33.3 | 42.5 |
| TMP92.36.286 | *18.2* | 14.5 | 18.7 | 17.6 | 22.1 | 26.0 | 42.3 | 61.0 |
| TMP92.93.1 |  |  |  |  |  |  |  |  |
| TMP93.36.257 |  |  | 3.2 |  | 5.8 |  |  |  |
| TMP95.12.147 |  |  |  |  |  |  |  |  |
| TMP99.62.1 |  |  |  |  |  |  |  |  |
| UALVP2 |  |  | 21.7 | 17.3 | 24.0 | 31.5 | 50.2 | 72.9 |
| UALVP49531 |  |  | 3.4 |  |  |  | 32.1 |  |
| UALVP51913 |  |  | 4.3 | 4.1 | 5.9 |  |  | 43.6 |
| UALVP6 |  |  |  |  |  | 21.3 | 33.8 | 50.1 |
| UALVP8502 | 21.3 | 23.8 | 15.9 | 14.4 | 20.2 | 28.9 |  |  |
| UAVLP8504 | 10.2 | 10.9 | 12.5 | 8.9 | 15.3 | 18.0 | 40.0 | 55.4 |
| UCMZ(VP)2008.001 |  |  |  |  |  |  |  |  |
| UCMZ(VP)2008.002 |  |  |  |  |  |  |  |  |
| UCMZ(VP)unnumbered | 15.0 | 8.0 | 10.5 | 8.1 | 11.8 | 23.8 | 47.2 | 57.6 |

| Specimen | W:pso/po | W:f/p | W:po/stf/sq | L:aso | L:pso | L:po | L:f | L:p | L:fp | T:f/p |
| --- | --- | --- | --- | --- | --- | --- | --- | --- | --- | --- |
| AMNH5450 | 45.4 | 39.2 | 35.7 | 10.1 | 15.5 | 24.5 | 41.3 | 41.0 | 82.3 | 20.0 |
| BMNH-R.-8673 |  | 62.2 |  |  |  |  |  |  |  | 44.6 |
| BMNH-R.-8674 |  | 53.1 |  |  |  |  |  |  |  | 38.2 |
| CMN1108A | 55.3 | 49.6 | 36.4 | 11.4 | 17.1 | 29.7 | 41.3 |  |  | 49.6 |
| CMN138 | 56.6 | 53.1 | 41.2 | 12.1 | 16.0 | 33.9 | 43.4 | 40.9 | 84.2 | 34.0 |
| CMN1594 |  | 57.2 | 42.5 |  |  |  |  | 51.1 |  | 30.6 |
| CMN2379 | 64.1 | 58.2 |  | 15.2 | 20.6 | *36.1* | 39.5 |  |  | 39.0 |
| CMN38428 | 67.9 | 70.8 | 55.6 | 17.2 | 23.1 | 36.0 | 55.4 | 63.7 | 119.1 | 40.0 |
| CMN515 | 58.5 | 57.5 | 44.3 | 9.4 | 20.2 | 35.8 | 45.5 | 48.0 | 93.5 | 35.0 |
| CMN8816 | 54.2 | 48.6 | 32.5 | 10.5 | 16.7 | 32.0 | 40.6 | *43.7* | 84.3 | 29.5 |
| ROM58311 |  | 69.6 |  | 12.2 | 23.3 | 24.0 | *41.4* | *54.7* | 82.6 | 42.1 |
| ROM803 | 56.7 | 53.4 |  | 9.4 | 16.6 | 34.3 |  |  |  |  |
| ROM53555 |  | 98.3 |  |  |  |  |  |  |  | 62.0 |
| TMP2000.26.1 |  | 95.3 |  | 22.1 | 28.3 | 49.1 |  |  |  | *63.8* |
| TMP2002.12.57 | *44.0* | *36.2* |  | 10.4 | 14.0 |  | 38.8 |  |  | 16.7 |
| TMP75.11.5 | *59.0* | 55.7 | 37.4 | 10.5 | 15.9 | 33.3 | *35.0* |  |  | *37.3* |
| TMP78.19.04 | *69.9* | *56.2* |  | 12.3 | 21.1 |  | *59.0* |  |  | 16.2 |
| TMP81.41.102 | *45.0* | *37.6* |  | *5.9* | 12.0 |  | 36.5 |  |  | 6.8 |
| TMP82.20.189 |  | 34.3 | 26.8 |  |  |  |  | 36.0 |  | 7.2 |
| TMP83.67.1 | 71.9 | 66.8 | 47.4 | 12.5 | 21.7 | 36.1 | *45.8* | *57.7* | *103.5* | 47.1 |
| TMP84.05.01 | 56.3 | 52.7 |  | 11.8 | 14.9 | 29.2 | 44.5 | 41.2 | 85.7 | 25.0 |
| TMP85.58.68 | *36.4* | 32.8 |  |  |  |  |  |  |  | 5.8 |
| TMP86.71.2 | *48.6* | *44.8* |  | 8.5 | 15.8 |  |  |  |  | 18.3 |
| TMP88.116.49 |  | *40.0* |  |  |  |  |  | 44.8 |  | 11.6 |
| TMP92.02.36 | 48.3 | 41.7 | 34.7 | 10.1 | 16.7 | 23.2 | 36.0 | *38.0* | 74.0 | 31.5 |
| TMP92.36.286 | 65.7 | 56.6 | 42.3 | 15.0 | 18.5 | 33.6 | 42.0 |  |  | 40.2 |
| TMP92.93.1 |  | *36.8* |  |  |  |  |  | 36.2 |  | 6.8 |
| TMP93.36.257 |  | *38.6* |  |  |  |  |  |  |  | 7.3 |
| TMP95.12.147 |  | 39.1 | 34.8 |  |  |  |  | 46.1 |  | 7.8 |
| TMP99.62.1 |  | 58.0 |  |  |  |  |  | 50.0 |  | 32.8 |
| UALVP2 | 85.9 | 81.3 | 59.9 | 17.9 | 22.2 | 42.4 |  | *67.6* | *116.3* | *62.2* |
| UALVP49531 |  | 32.7 |  |  |  |  |  | 38.6 |  | 6.6 |
| UALVP51913 | 46.0 | *41.2* |  | 10.0 | 11.9 |  |  |  |  | 7.0 |
| UALVP6 | 55.9 | 55.8 |  | 13.6 | 23.1 |  | *47.0* |  |  | 40.8 |
| UALVP8502 |  | *56.0* |  | 14.2 | 18.0 | 33.0 | 42.4 |  |  | 37.6 |
| UAVLP8504 | *64.4* | 62.0 |  | 12.0 | 18.0 |  | 48.8 |  |  | 30.8 |
| UCMZ(VP)2008.001 |  | 33.6 |  |  |  |  |  | 36.6 |  | 7.8 |
| UCMZ(VP)2008.002 |  | 30.8 |  |  |  |  |  | 31.2 |  | 6.8 |
| UCMZ(VP)unnumbered | 57.2 | 53.0 |  | 8.2 | 15.4 | 37.8 | 48.4 |  |  | 53.2 |

Measurements are in mm. Italicized measurements are approximate. Abbreviations: **H:n/n**, height of the sutural surface at the contact of the nasals; **H:n/prf**, height of the sutural surface at the contact of the nasal and prefrontal; **H:prf/aso**, height of the sutural surface at the contact of the prefrontal and anterior supraorbital; **H:aso/pso**, height of the sutural surface at the contact of the anterior supraorbital and posterior supraorbital; **H: pso/po**, height of the sutural surface at the contact of the posterior supraorbital and postorbital; **T:fp**, thickness of the frontoparietal; **L:aso**, length of the supraorbital suture; **L:pso**, length of the posterior supraorbital suture; **L:po**, length of the postorbital suture; **L:f**, length of the frontal; **L:p**, length of the parietal; **L:fp**, length of the frontoparietal; **W:n/prf**, width between the nasal/prefrontal sutural contacts; **W:prf/aso**, width between the aso/pso supraorbital sutural contacts; **W:aso/pso**, width between anterior and posterior supraorbital sutural contacts; **W:pso/po**, width between the posterior supraorbital and postorbital sutural contacts; **W:po/stf/sq**, width between the contacts of postorbital suture and the supratemporal fenestrae or the squamosal suture if fenestrae are closed.
