# Supplementary material for: Cranial Ontogeny in Stegoceras validum (Dinosauria: Pachycephalosauria): A Quantitative Model of Pachycephalosaur Dome Growth and Variation
Source: PLoS One. 2011 Jun 29;6(6):e21092. doi: 10.1371/journal.pone.0021092 (PMC3126802; doi:10.1371/journal.pone.0021092)
Supplement: Text S1 — Description of measurements. (DOC) [file pone.0021092.s002.doc]

**Text S1. Description of measurements.**

All measurements were taken between homologous morphological landmarks. Most are positioned at the dorsal margin of the contact between sutural surfaces for the peripheral skull bones on the frontoparietal. These landmarks are as follows: n/n, n/prf, prf/aso, aso/pso, pso/po, po/stf/sq. The widths, heights, and length of the sutural surfaces were all taken between the corresponding landmarks. The length of the frontal, parietal and frontoparietal were measured ventrally along the midline between landmarks located at the anterior edge of the frontoparietal at the sutural contact of the nasals, along the midline at the contact between the frontals and parietal in the endocranial fossa, and at the posterior edge of the dome along the midline. Frontoparietal thickness was measured using proportional calipers between landmarks located within the endocranial fossa at the contact of the frontals and parietal and on the dorsal surface of the frontoparietal at the contact of the frontals and parietal. All other measurements were done using standard digital callipers. Locations of each landmark are shown in Figure 3. Abbreviations: **aso**, anterior supraorbital; **n**, nasal; **prf**, prefrontal; **po**, postorbital; **pso**, posterior supraorbital; **sq**, squamosal; **stf**, supratemporal fenestrae.
