# Supplementary material for: Cranial Ontogeny in Stegoceras validum (Dinosauria: Pachycephalosauria): A Quantitative Model of Pachycephalosaur Dome Growth and Variation
Source: PLoS One. 2011 Jun 29;6(6):e21092. doi: 10.1371/journal.pone.0021092 (PMC3126802; doi:10.1371/journal.pone.0021092)
Supplement: Text S2 — HRCT methods. (DOC) [file pone.0021092.s003.doc]

**Text S2. HRCT methods.**

**University of Texas High-Resolution X-ray CT Facility**

Scans of the frontoparietal of *Ornatotholus browni* (AMNH 5450; Late Cretaceous Dinosaur Park Fm, Alberta Canada, collected by Barnum Brown, 1913) for Dr. Mark Goodwin of the Museum of Paleontology, The University of California at Berkeley. Specimen scanned by Matthew Colbert 23 October 2003.

1024x1024 16-bit TIFF images. II, 180 kV, 0.133 mA, no filter, empty container wedge, no offset, slice thickness 2 lines (= 0.1066 mm), S.O.D. 155 mm, 1000 views, 2 samples per view, inter-slice spacing 2 lines (= 0.1066 mm), field of reconstruction 50 mm (maximum field of view 50.79406 mm), reconstruction offset 5000, reconstruction scale 800. Acquired with 15 slices per rotation. Reconstructed with beam-hardening correction: 0.0, 0.8, 0.15. Rotation correction processing done by Rachel Racicot using IDL routine “DoRotationCorrection”. Total slices = 824.

Scans of an incomplete skull of *Stegoceras sp.* (TMP 84.05.01; Judith River Fm, Upper Cretaceous; Steveville Area, AB) for Dr. Mark Goodwin of the Museum of Paleontology, the University of California at Berkeley. Specimen scanned by Matthew Colbert 4 November 2003.

1024x1024 16-bit TIFF images. II, 180 kV, 0.133 mA, no filter, empty container wedge, no offset, slice thickness 1 line (= 0.1095 mm), S.O.D. 315 mm, 1200 views, 2 samples per view, inter-slice spacing 1 line (= 0.1095 mm), field of reconstruction 101 mm (maximum field of view 103.2266 mm), reconstruction offset 8000, reconstruction scale 650. Acquired with 27 slices per rotation. Reconstructed with beam-hardening correction: 0.0, 0.6, 0.25, 0.1. Rotation correction processing done by Rachel Racicot using IDL routine “DoRotationCorrection”. Total slices = 945.

Scans of a partial skull of *Stegoceras validum* (ROM 53555; Ornithischia, Pachycephalosauridae; Upper Cretaceous, Campanian; Belly River Group, Dinasaur Park Fm?; Red Deer River, near Dinosaur Provincial Park) for David Evans of the Royal Ontario Museum. Specimen scanned by Matthew Colbert 13 May 2010.

1024x1024 16-bit TIFF images. P250D, 450 kV, 1.3 mA, small spot size, 1 brass filter, air wedge, 190% offset, integration time 32 ms, slice thickness = 0.25 mm, S.O.D. 585 mm, 1600 views, 1 ray per view, 1 sample per view, inter-slice spacing = 0.23 mm, field of reconstruction 232.6705 mm (maximum field of view 236.1214 mm. Field of view reported on scan form corresponds to a corrected value after applying a correction of 1.010942%. Field of view used in ACTIS equals 230.1522 mm. Reported maximum field of view reflects uncorrected value. This scan was originally archived with an incorrectly reported field of view, which was corrected in August, 2010.), reconstruction offset 5800, reconstruction scale 6000. Streak- and ring-removal processing done by Satomi Ishihama based on correction of raw sinogram data using IDL routines “RK_SinoDeStreak” with parameter “cutoff=0.08” and “RK_SinoRingProcSimul” with parameters “bestof5=11” (applied to some slices) and “nuke_INDs=[453-455, 519-521]” (applied to slices 208, 259-264). Reconstructed with rotation angle = 3 degrees. Post-reconstruction ring correction done by Satomi Ishihama using UTCT ring correction program with the parameters “oversample=3.0, binwidth=19, sectors=60” for slices 208, 259-264, and with parameters “oversample=3.0, binwidth=23, sectors=1” for select slices. Total final slices = 376.
